# A large chromosomal inversion shapes gene expression in seaweed flies (*Coelopa frigida*)

**DOI:** 10.1002/evl3.260

**Published:** 2021-10-07

**Authors:** Emma L. Berdan, Claire Mérot, Henrik Pavia, Kerstin Johannesson, Maren Wellenreuther, Roger K. Butlin

**Affiliations:** ^1^ Department of Marine Sciences University of Gothenburg Gothenburg SE‐40530 Sweden; ^2^ Département de Biologie, Institut de Biologie Intégrative et des Systèmes (IBIS) Université Laval Québec QC G1V 0A6 Canada; ^3^ The New Zealand Institute for Plant and Food Research Ltd. Nelson 7010 New Zealand; ^4^ School of Biological Sciences University of Auckland Auckland 1010 New Zealand; ^5^ Ecology and Evolutionary Biology, School of Biosciences University of Sheffield Sheffield S10 2TN United Kingdom

**Keywords:** Chromosomal evolution, gene expression, genetic architecture, population genomics

## Abstract

Inversions often underlie complex adaptive traits, but the genic targets inside them are largely unknown. Gene expression profiling provides a powerful way to link inversions with their phenotypic consequences. We examined the effects of the *Cf‐Inv(1)* inversion in the seaweed fly *Coelopa frigida* on gene expression variation across sexes and life stages. Our analyses revealed that *Cf‐Inv(1)* shapes global expression patterns, most likely via linked variation, but the extent of this effect is variable, with much stronger effects in adults than larvae. Furthermore, within adults, both common as well as sex‐specific patterns were found. The vast majority of these differentially expressed genes mapped to *Cf‐Inv(1)*. However, genes that were differentially expressed in a single context (i.e., in males, females, or larvae) were more likely to be located outside of *Cf‐Inv(1)*. By combining our findings with genomic scans for environmentally associated SNPs, we were able to pinpoint candidate variants in the inversion that may underlie mechanistic pathways that determine phenotypes. Together the results of this study, combined with previous findings, support the notion that the polymorphic *Cf‐Inv(1)* inversion in this species is a major factor shaping both coding and regulatory variation resulting in highly complex adaptive effects.

Chromosomal inversions, pieces of the chromosome that have been flipped 180°, are structural variants that may encompass hundreds of genes but segregate together as a single unit due to suppressed recombination. Recombination between arrangements (i.e., orientations) is reduced in heterokaryotypes but proceeds freely in both homokaryotypes. This reduced recombination can shield adaptive allelic combinations from gene flow, facilitating evolutionary processes such as local adaptation (Kirkpatrick and Barton 2006; Schaeffer 2008; Twyford and Friedman 2015; Westram et al. 2021), sex chromosome evolution (Peichel et al. 2004; Lemaitre et al. 2009; Knief et al. 2017). and speciation (Kirkpatrick and Barton 2006; Hoffmann and Rieseberg 2008; Lowry and Willis 2010; Ayala et al. 2013; Twyford and Friedman 2015; Wellenreuther and Bernatchez 2018). Inversions underlie major phenotypic polymorphisms in a wide variety of taxa, such as male reproductive morphs in the ruff, *Philomachus pugnax* (Küpper et al. 2016; Lamichhaney et al. 2016), and Müllerian mimicry wing patterns in the butterfly *Heliconius numata* (Joron et al. 2011). However, the reduced effective recombination that allows inversions to have these profound effects may also limit the ability to detect signatures of selection due to extreme linkage disequilibrium. This encumbers detection of the mechanistic pathways that generate phenotypic effects as well as identification of the underlying adaptive variants.

The linkage disequilibrium in inversions presents many challenges to identify adaptive variation. Because effective recombination between arrangements is reduced, forward genetic approaches such as Quantitative Trailt Loci (QTL) mapping or genome wide association studies are not feasible for variation that is fixed between arrangements (Noor et al. 2001). Additionally, the reduced recombination and effective population size within the inverted region facilitate the accumulation of neutral and deleterious variation (Berdan et al. 2021), increasing divergence between the arrangements and increasing the likelihood of detecting phenotype or environment associations with noncausative loci. Finally, larger inversions, such as the *lnv4m* inversion in *Zea mays*, may contain hundreds of genes that affect a wide variety of phenotypes that vary in their selective pressures (Crow et al. 2020).

Transcriptomic analysis offers a way to address the links between individual loci and the phenotypic effects of an inversion by uncovering functionally important variation in a way that is not hindered by linkage disequilibrium in natural populations or recombination suppression in controlled crosses. This is because (1) the phenotypic effects of inversions might be underlain in part by changes in gene expression, and (2) overlap between differentially expressed genes (from transcriptomic studies) and outlier SNPs (from genomic studies, i.e., loci associated with adaptive traits or ecological factors) facilitates the identification of candidate genes (Renaut et al. 2011; Kozak et al. 2014; Pardo‐Diaz et al. 2015).

There are three major (nonexclusive) ways that inversions may affect gene expression. First, inversions may modify the epigenetic environment near their breakpoints (Lupiáñez et al. 2015; Shanta et al. 2020). Second, breakpoints may change the relative positions of genes and their transcription regulators, changing expression patterns (Lettice et al. 2011; Lavington and Kern 2017). Third, the linked variation within an inversion can contain *cis*‐ or *trans*‐acting regulatory elements that can evolve independently in the two arrangements due to suppressed recombination between them (Huang et al. 2015; Fuller et al. 2016; Said et al. 2018; Crow et al. 2020). As variants within inversions are highly linked, it is difficult to distinguish between *cis*‐regulation and *trans*‐acting loci in linkage disequilibrium with their targets. Here, we focus on whether differentially expressed loci are contained within the inverted region (hereafter referred to as *cis*‐regulated for karyotype) or located in other areas of the genome (hereafter referred to as *trans*‐regulated for karyotype). Overall, these effects on gene expression can be fixed, vary across life stages or sexes, or show plastic responses to the environment.

In this study, we investigated the effect of a large inversion on expression variation and combined this analysis with previously published population genomic data to identify putatively adaptive loci. We use the seaweed fly, *Coelopa frigida*, which inhabits “wrackbeds” (accumulations of decomposing seaweed) on North Atlantic shorelines. This fly has an inversion polymorphism on chromosome I (*Cf‐Inv(1)* spanning 60% of chromosome 1 and 10% of the genome, corresponding to about 25 MB) (Merot et al. 2020a). *Cf‐Inv(1)* has two highly diverged arrangements, termed α and β, resulting from three overlapping inversions (Aziz 1975). The inversion influences multiple measurable traits in males such as mating success (Aziz 1975; Day et al. 1983; Edward 2008), development time (Butlin et al. 1982; Gilburn and Day 1994; Mérot et al. 2020b), longevity (Butlin and Day 1985a), and adult size (Butlin et al. 1982; Mérot et al. 2018). Of these, size is the trait where the inversion has the strongest effect; αα males are approximately threefold heavier than ββ males (Berdan et al. 2018b). This is mirrored in development time with αα males taking significantly longer to reach adult eclosion than ββ males (Butlin et al. 1982). Conversely, female phenotype is mostly unaffected by karyotype, although there are small effects on size (Butlin and Day 1985a; Mérot et al. 2020b). The sex difference in the effect of the inversion indicates a particular role for gene expression as males and females largely share the same genome.

*Cf‐Inv(1)* is under multiple forms of natural and sexual selection. The inversion is polymorphic in all investigated natural populations to date and maintenance of the polymorphism is mostly through balancing selection caused by strong overdominance of the heterokaryotype (Butlin 1983; Day et al. 1983; Butlin and Day 1984, 1989; Mérot et al. 2018). This overdominance is due to increased survival of heterokaryotypic larvae, particularly when flies reach high densities, combined with trade‐offs in survival and fecundity between the two homokaryotypes (Butlin et al. 1982; Butlin and Day 1985a, 1985b; Mérot et al. 2020b). In general, αα individuals (especially males) enjoy higher mating success due to their larger size and higher fecundity (in females) but show lower larval survival rates and develop more slowly, whereas the converse is true for ββ individuals. Several lines of evidence indicate that *Cf‐Inv(1)* is also under directional natural selection that modifies the equilibrium frequency attained due to overdominance. The frequency of the different arrangements varies depending on the seaweed they are found on, with kelp favoring α and bladderwrack favoring β (Day et al. 1983; Mérot et al. 2018) and populations are locally adapted to their own seaweed composition (Wellenreuther et al. 2017; Berdan et al. 2018a). Finally, other abiotic characteristics such as wrackbed depth and temperature also influence the relative fitness of the two homokaryotypes (Day et al. 1983; Mérot et al. 2018). However, the specific phenotypes associated with these fitness differences remain unknown.

We collected *C. frigida* from natural populations (Figure 1A) and examined how *Cf‐Inv(1)* shaped gene expression across sexes and life stages. Specifically, our study had three major goals: (1) To examine the effect of karyotype on global expression patterns in adults and larvae and to determine if these effects are common across sexes and life stage or context specific, (2) To ascertain if these genes are *cis*‐ or *trans‐*regulated with respect to *Cf‐Inv(1)*, and (3) To identify putative adaptive variation within the inversion and connect this with ecological niche differences between karyotypes.

**Figure 1:**
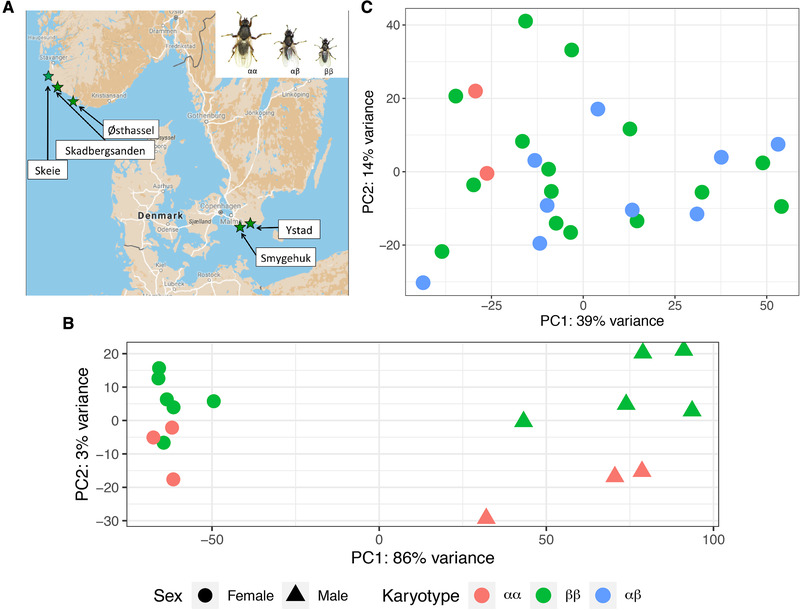
Variation in expression differs across life stages. (**A)** Map of Norway, Denmark, and Sweden showing the populations sampled. The inset shows size variation in males as a function of karyotype. (**B)** Principal component analysis (PCA) of expression variation in adults. Points are colored by karyotype (αα, red; ββ, green) and shaped according to sex (female, circle; male, triangle). (**C)** PCA of expression variation in larvae; all samples are pools of three larvae of unknown sex colored by karyotype (αα, red; αβ, blue; ββ, green). Both Figures 1B and 1C are based on the top 500 transcripts with the highest variance.

## Results and Discussion

### SEQUENCING AND TRANSCRIPTOME ASSEMBLY

To study gene expression variation associated with sex, life stage, and karyotypes of the inversion, we sequenced RNA from 17 adult individuals and 28 larval pools. We used part of this dataset to create the first reference transcriptome for *C. frigida*. Our final transcriptome assembly contained 35,999 transcripts with an N50 of 2155 bp, a mean length of 1092 bp, and a transrate score (Smith‐Unna et al. 2016) of 0.4097. The transcriptome has good coverage, it has a BUSCO score of 86.6% (2393 complete and single copy [85.5%], 31 complete and duplicated [1.1%], 190 fragmented [6.8%], and 185 missing [6.6%]), and 95% of the reads mapped back to the transcriptome (Simão et al. 2015). Using the trinotate pipeline (Trinotate.github.io), we were able to annotate 14,579 transcripts (40%) from the transcriptome. This high‐quality transcriptome will provide a useful resource for any future work on this and related species, provide a much‐needed functional map for better understanding the regulation of genes across life stages and sexes, and facilitate the identification of functional phenotypes that correspond to inversions.

### THE EFFECT OF *Cf‐Inv(1)* ON GENE EXPRESSION IS STRONG BUT VARIABLE

In adults, karyotype was the second strongest factor explaining expression variation. Decomposing adult expression variation into a principal component analysis (PCA), we found that the PC1, explaining 86% of the variance, separated males and females, whereas PC2, explaining 3% of the variance, separated αα and ββ in both males and females (Fig. 1B). This strong sex difference was mirrored in our differential expression analysis; a total of 3526 out of 26,239 transcripts were differentially expressed between the sexes with a strong bias toward increased expression in males (68% of differentially expressed genes upregulated in males; Fig. S1).

Sex modulated the effects of *Cf‐Inv(1)* on global expression patterns. When combining the sexes, 304 out of 26,239 transcripts were differentially expressed between αα and ββ (Fig. S2). A distance matrix analysis revealed that (1) average similarity between pairs of females was higher than between pairs of males and (2) males clustered by karyotype, whereas females did not (Fig. S3). Due to these strong differences, we chose to run separate analyses for the sexes instead of analyzing the interaction term from our main model. Comparing homokaryotypic sex groups separately (αα vs. ββ) revealed that more than double the number of differentially expressed genes were found in males compared to females (801 vs. 340; Figs. S4 and S5). Note that males and females expressed a similar number of genes (e.g., had a total read count across all samples >10 for 21,149 and 21,579 genes, respectively). There was substantial overlap between differentially expressed genes in the two sexes with the highest proportion of unique differentially expressed genes found in males (Fig. S6). The phenotypic effects of *Cf‐Inv(1)* are also strongly sex specific. This is likely due to sexual selection that, in *C. frigida*, has partly evolved in response to strong sexual conflict over reproduction, specifically mating rate (Crean and Gilburn 1998; Dunn et al. 1999). This sexual conflict over mating rates has selected for sexual dimorphism in some of the external phenotypic traits used for mating, notably size and cuticular hydrocarbon composition (Enge et al. 2021). Larger males (usually αα) are more successful in obtaining copulations and resisting the rejection responses that females use to prevent male mountings. The *Cf‐Inv(1)* inversion has a large impact on a range of traits: the morphology of males (Butlin et al. 1982; Gilburn and Day 1994), development time (Butlin and Day 1984; Mérot et al. 2020b), and the composition of cuticular hydrocarbons (Enge et al. 2021). It was thus no surprise that males showed a larger gene expression difference between karyotypes compared to females.

Surprisingly, *Cf‐Inv(1)* was not a primary factor explaining variance in larval gene expression. A PCA in larvae found that the first two PCs (explaining 52% of the variance) did not separate samples based on karyotype (Fig. 1C), instead a separation by population was observed (Fig. S7). We ran an additional PCA on the larval data using only the Skeie population (the only population with all three karyotypes), to remove population variation. The first two PCs (explaining 67% of the variance) together separated the karyotypes, albeit weakly (Fig. S8).

To formally test the role of karyotype in partitioning variation, we ran a PERMANOVA on Manhattan distances for each subgroup (i.e., males, females, and larvae; Table S2) (Dixon 2003). As different tests had different sample sizes, we concentrated on *R* ^2^ values (sum of squares of a factor/total sum of squares). Males and females had the highest *R* ^2^ values (0.2464 and 0.153, respectively) followed by all adults and larvae (0.084 and 0.073, respectively). These results match our qualitative observations that karyotype explains the largest proportion of variance in adult males followed by adult females and then larvae. However, the comparison of our combined adult model with the sex‐specific models shows that separating sex is critical for quantifying the effect of karyotype. Thus, the superficial appearance of inversion having less influence on larval gene expression may be because larval sex was not determined.

Further dissecting differential expression in our full larval dataset corroborated our qualitative observations. Because we had three genotypes in larvae (αα, αβ, and ββ), we ran three different contrast statements (αα vs. ββ, αβ vs. ββ, and αα vs. αβ). When comparing expression in ββ versus αβ, we found that 23 out of 15,859 transcripts were differentially expressed and most of these (74%) were upregulated in αβ (Fig. S9). Comparing expression in αα versus. ββ, we found 29 out of 15,859 transcripts to be differentially expressed and most of these (83%) were upregulated in ββ (Fig. S10). Comparing expression in αα versus αβ, we found six out of 15,859 transcripts to be differentially expressed and most of these (83%) were upregulated in αβ. There was some overlap between these three contrasts (Fig. S11). We compared expression patterns of our significantly and differentially expressed transcripts across all three contrasts. Using unadjusted *P*‐values, we classified transcripts as additive (αα > αβ > ββ or αα < αβ < ββ), underdominant (αβ < αα and αβ < ββ), overdominant (αβ > αα and αβ > ββ), β‐dominant (αβ = ββ, αβ < αα, or αβ > αα), α‐dominant (αβ = αα, αβ < ββ, or αβ > ββ), or unknown (see Table S3). We found 11 additive transcripts (nine located within *Cf‐Inv(1)*), one underdominant transcript, no overdominant transcripts, 19 β‐dominant transcripts (nine located within *Cf‐Inv(1)*), and 24 α‐dominant transcripts (10 located within *Cf‐Inv(1)*). The vast majority of differentially expressed genes showed some form of dominance or were additive (98%) indicating that αα and ββ generally represent expression extremes in this system. In *C. frigida*, heterokaryotype overdominance does not seem to be linked to overdominant expression as is found in many examples of heterosis (Chen 2013). Instead, data from within‐ and between‐population crosses suggest that the observed overdominance of the heterokaryotype is actually associative overdominance caused by the masking of deleterious alleles (Ohta 1971; Butlin and Day 1985b). Under this model, the null expectation would be additive expression or dominant expression (in cases where natural selection in heterokaryotypes has selected for the allele with fewer deleterious mutations), which is what we observe.

Compared to the adults, a lower proportion of transcripts were significantly differentially expressed between αα versus ββ larvae (1.16−3.05% in adults, 0.2% in larvae). In addition to pooling sexes in larvae, there are several other features of our experimental design that could have contributed to the reduced effect in larvae. First, our crossing design generated only two αα larval pools compared to 10 αβ larval pools and 16 ββ larval pools. Thus, our contrasts that included αα had lower power. We also generated more variation in our larval samples compared to our adults as we crossed both within and between populations, whereas adults were all single population origin. It is possible that this variation made detection of differentially expressed genes more difficult. However, our results still clearly suggest that the effect of *Cf‐Inv(1)* on gene expression is strongly conditional on life stage and sex.

### ALLELE‐SPECIFIC EXPRESSION WITHIN *Cf‐Inv(1)*

**Figure 2:**
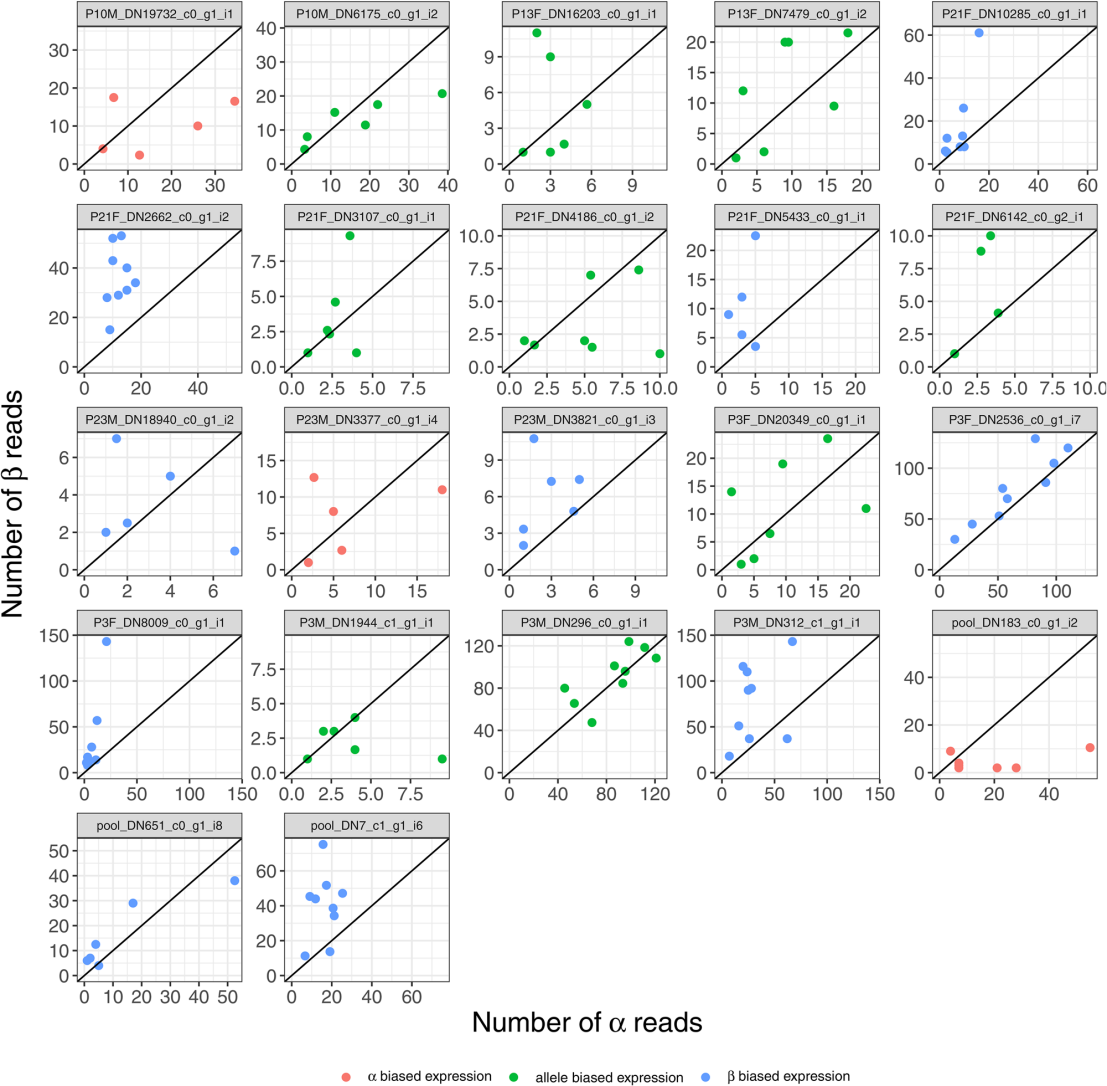
Patterns of allele specific expression (ASE). Each plot is for a single transcript where each dot represents a single αβ individual averaged over all SNPs in that transcript. A 1:1 line is provided for context. Colors indicate the expression pattern: α‐biased expression, red; β‐biased expression, blue; allele‐biased expression, green. Note that only transcripts with data for five or more individuals are shown here. The full dataset is shown in Figure S12.

Beyond quantitative differences of expression, genes within *Cf‐Inv(1)* were also characterized by allele‐specific expression (ASE) in heterokaryotypes. Concentrating on loci that were fixed between arrangements, we retained 315 out of 619,424 SNPs found across 113 transcripts all located within *Cf‐Inv(1)*. Using the ASEP package (Fan et al. 2020) with our nine αβ larval pools, a total of 30 out of 113 transcripts had significant ASE (Fig. S12). We compared this with our complete differential expression results and found that only a single transcript overlapped between the two. For each of thes transcripts, we averaged read depth across all SNPs per transcript, per individual. We classified them as “α‐biased expression” if >50% of the larval pools (i.e., replicates) had ≥55% α‐allele reads and as “β‐biased expression” if >50% of the larval pools had ≥55% β‐allele reads. To explore our cutoff choice, we generated 10,000 binomial trails with a 1:1 outcome using the mean read depth of our data (44). Using a 55% cutoff, we expect a false positive error rate of approximately 22.6% compared to a 44% error rate using a 50% cutoff. Given a 22.6% error rate and six replicates (our average), the estimated error rate for our classification scheme is 0.31% (compared to 3.3% with a 50% cutoff for reads). We thus considered 55% a conservative cutoff. If neither of these conditions was met, that is, the direction was inconsistent, we simply labeled them as “allele‐biased expression.” We found five transcripts that showed “α‐biased expression,” 12 transcripts that showed “β‐biased expression,” and 13 transcripts that showed “allele‐biased expression” (Fig. S12; transcripts with data for five or more individuals are shown in Fig. 2). There were no significant gene ontology (GO) terms for any of these groupings. Two interesting patterns emerge from these data. First, allele‐biased expression, when present, seems to be relatively consistent across populations. Our αβ larvae resulted from crosses within and between populations yet we found consistent ASE patterns in 56% of our ASE transcripts. Second, differentially expressed genes showed no propensity toward ASE as only one out of 30 ASE genes showed significant differential expression in any of our analyses and most showed close to zero differential expression (e.g., from the combined adult αα vs. ββ comparison, the mean absolute log2fold change was 0.75). This indicates that ASE may be evolving somewhat independently from differential expression. Overall, these results demonstrate that there is allele‐biased expression within inversions but the extent of this phenomenon and the resulting phenotypic implications remain unknown.

### GENES WITH CONSTANT KARYOTYPE EFFECTS ARE OVERWHELMINGLY *CIS*‐REGULATED WHILE GENES WITH CONDITIONAL EFFECTS ARE MORE LIKELY TO BE *TRANS*‐REGULATED

**Figure 3:**
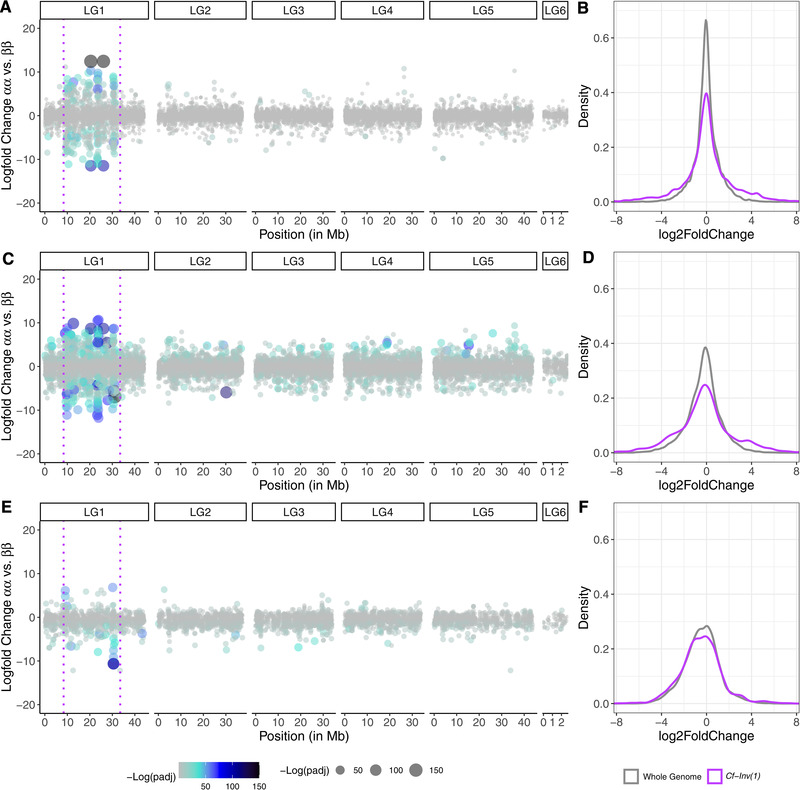
Differential expression is mostly *cis*‐regulated for karyotype. Differentially expressed transcripts along the genome in (A) females, (C) males, and (E) larvae. *Y*‐axes denote logfold change between αα and ββ and *x*‐axes denote position in megabases. The dotted magenta lines denote the location of *Cf‐Inv(1)*. Note that position in LG6 is not to scale with the other linkage groups for presentation. Each dot is a single transcript and both color and size denote the –log (*P*‐value) after false discovery rate correction. Next to each graph are density plots of log2fold changes for αα versus ββ comparisons for all loci in the genome (colored gray) and just loci within *Cf‐Inv(1)* (colored magenta) for each group: females (B), males (D), and larvae (F). Negative values indicate higher expression in ββ.

Most of the differentially expressed genes mapped within *Cf‐Inv(1)* (Fig. 3). For adults, 12.8% of transcripts tested for differential expression were found within *Cf‐Inv(1)* (Table 1), which is approximately what might be expected, as *Cf‐Inv(1)* comprises 10.5% of the genome (Mérot et al. 2021). However, 80.6% of the transcripts that were differentially expressed between αα and ββ (with the sexes combined) were found within *Cf‐Inv(1)* (odds ratio = 28.3). Looking at this in a different way, 7.2% of the transcripts within the inversion were differentially expressed between karyotypes compared to 0.3% of genes in the collinear region. No differentially expressed genes were found immediately adjacent to the estimated breakpoints (Mérot et al. 2021). The closest two loci to the distal and proximal breakpoints (P23M_ DN102_c0_g1_i8 and P10M_ DN23301_c0_g1_i1) were found 73 and 410 kb away, respectively. Furthermore, the distribution of all differentially expressed genes within *Cf‐Inv(1)* shows no clustering near the breakpoints (Fig. S13). These general patterns indicate that the breakpoints themselves (and any subsequent chromatin changes) are unlikely to be responsible for these expression patterns. This is in line with other studies in *Drosophila* (Fuller et al. 2016; Said et al. 2018) and maize (Crow et al. 2020) that implicate linked variation rather than the breakpoints themselves.

**Table 1:** Location of differentially expressed transcripts

Location	Tested transcripts	Differentially expressed between αα and ββ	Differentially expressed between males and females
LG1 (excluding *Cf‐Inv(1)*)	10.6%	3.1%	12.0%
*Cf‐Inv(1)*	12.8%	80.5%	11.6%
LG2	18.6%	2.7%	18.8%
LG3	16.6%	3.8%	17.4%
LG4	18.0%	3.8%	19.0%
LG5	17.5%	3.4%	17.6%
LG6	1.6%	0.0%	0.7%
Other Scaffolds	4.3%	2.7%	2.9%

Proportion of differentially expressed or tested transcripts is shown as a percentage located within different linkage groups or inversions. The LG1 category excludes *Cf‐Inv(1)*. The “Other Scaffolds” category sums across 340 scaffolds that could not be incorporated into existing linkage groups (for details, see Mérot et al. 2021). The total number of transcripts represented by each group is as follows: 25,320 (tested transcripts), 293 (DE between αα and ββ), and 3411 (DE between males and females).

When decomposing the sexes, the *cis*‐effect was much stronger in females than males as 78% of differentially expressed genes in females (odds ratio = 24.2) were found within *Cf‐Inv(1)* compared to 44.5% in males (odds ratio = 5.5; Fig. 3A, C). For larvae, we combined the ββ versus αβ, αα versus αβ, and αα versus ββ contrasts as so few differentially expressed transcripts were found (a combined total of 55 transcripts). Of these, 52.8% were found within *Cf‐Inv(1)* (odds ratio = 7.6). This effect is visible when comparing density plots for log2fold changes from αα versus ββ comparisons from the entire genome to within *Cf‐Inv(1)* (Fig. 3B, D, F). Here, we see two trends. First the whole genome density plots for both males (Fig. 3D) and larvae (Fig. 3F) are much flatter and left shifted than the density plot for females (Fig. 3B). Second, for all three groups the density plots for genes within *Cf‐Inv(1)* are wider and more left shifted. All of these differences were significant with two sample Kolmogorov‐Smirnov tests but the effect was weaker when comparing the whole genome versus within *Cf‐Inv(1)* in larvae (Table S4). Compared to karyotype, the effect of sex showed no pattern of localization. Instead, transcripts differentially expressed between males and females in adults closely matched the null distribution of tested transcripts (Table 1).

The fact that most of the differentially expressed genes were *cis*‐regulated for karyotype but not for sex effects is consistent with the idea that gene expression presents a major substrate for evolutionary change. Other recent studies of expression variation between karyotypes have also found strong *cis*‐effects (Fuller et al. 2016; Lavington and Kern 2017; Said et al. 2018). Allele‐biased expression is expected under *cis*‐regulation so these results are concordant with our ASE analysis (Knight 2004). Interestingly, the group where the strongest phenotypic differences are present (males) showed more *trans*‐effects of *Cf‐Inv(1)*. Furthermore, differentially expressed transcripts that were shared between analyses were more likely to be located within *Cf‐Inv(1)*. Of transcripts significant in both the male and female comparisons, 92.3% map to *Cf‐Inv(1)* compared with 59.8% of transcripts unique to the female analysis and 29% of transcripts unique to the male analysis. Overall, these results suggest that the “base” effect of the inversion might be mostly *cis*‐regulated, whereas conditional effects may be more likely *trans*. *Cis‐*regulatory elements are physically linked to the genes whose expression they control and thus tend to influence one or a few gene targets, often in specific tissues or at specific times, whereas more distant *trans*‐factors can control the expression of many genes. Thus, *trans*‐control of conditional effects in inversions may evolve more easily due to cascading effects. This is in line with evidence suggesting *trans*‐regulation may also be important for environment‐dependent changes in gene expression (Snoek et al. 2017; Signor and Nuzhdin 2018). Our results highlight the importance of comparing the effects of inversions on gene expression in multiple contexts (i.e., sexes, life stages).

### PROCESSES AFFECTED BY *Cf‐Inv(1)* INCLUDE METABOLISM AND DEVELOPMENT

To be able to connect changes in expression with the phenotypic effects of *Cf‐Inv(1)*, we first tested for enrichment of GO categories in differentially expressed genes between karyotypes and sexes (Table 2). We found 16 significantly enriched GO terms across all of our tests but removed one GO term as it was supported by a single transcript. The 15 remaining terms can be found in Table 2. The three terms associated with karyotype related to development (adult chitin‐based cuticle development) and metabolism/energy storage (digestion, positive regulation of triglyceride lipase activity). Unsurprisingly, the majority of the terms associated with sex differences were related to the production of gametes (e.g., sperm axoneme assembly, germ‐line stem cell division).

**Table 2:** Significantly enriched Gene Ontology terms

GO ID	Term	Annotated	Significant	Expected	elimF	Adjusted *P*‐value	Analysis	Additional analyses where significant
GO:0003341	Cilium movement	59	35	10.14	1.00 × 10^–7^	0.0003617	Sex	
GO:0006030	Chitin metabolic process	96	32	16.5	8.60 × 10^–5^	0.084835091	Sex	
GO:0006270	DNA replication initiation	26	18	4.47	6.30 × 10^–7^	0.00162765	Sex	
GO:0007288	Sperm axoneme assembly	15	11	2.58	2.60 × 10^–6^	0.0047021	Sex	
GO:0007305	Vitelline membrane formation involved in chorion‐containing eggshell formation	19	15	3.27	6.20 × 10^–9^	3.36 × 10^–5^	Sex	
GO:0007586	Digestion	99	10	1.03	7.00 × 10^–8^	0.00075957	Adult αα vs. ββ	Female αα vs. ββ, Larvae αα vs. ββ
GO:0008365	Adult chitin‐based cuticle development	8	7	0.25	1.90 × 10^–10^	2.06 × 10^–6^	Male αα vs. ββ	Sex
GO:0030720	Oocyte localization involved in germarium‐derived egg chamber formation	11	8	1.89	7.60 × 10^–5^	0.0824676	Sex	
GO:0034587	piRNA metabolic process	22	13	3.78	1.20 × 10^–5^	0.018601714	Sex	
GO:0035082	Axoneme assembly	67	40	11.51	6.10 × 10^–9^	3.36 × 10^–5^	Sex	
GO:0042078	Germ‐line stem cell division	29	14	4.98	0.00011	0.0994675	Sex	
GO:0060294	Cilium movement involved in cell motility	12	9	2.06	1.70 × 10^–5^	0.023058375	Sex	
GO:0061365	Positive regulation of triglyceride lipase activity	5	4	0.15	4.20 × 10^–6^	0.0227871	Male αα vs. ββ	
GO:1905349	Ciliary transition zone assembly	6	6	1.03	2.60 × 10^–5^	0.031347333	Sex	

Listed are: the GO ID, the term, the number of transcripts annotated with that term in the testing set, the number of these transcripts that were differentially expressed, the expected number of transcripts, the *P*‐value from the elim model with the Fisher's exact test, the adjusted *P*‐value, the analysis where the term was significant, and other analyses where the same term was significant. If a term was significant in multiple analyses, we show the data from the most significant test and list that one in the analysis column.

We also investigated the impact of *Cf‐Inv(1)* at the level of pathways by testing for polygenic expression patterns using Signet library (Gouy et al. 2017) and the KEGG pathway database (Kanehisa et al. 2017). We identified a number of gene subnetworks within biological pathways that show differential expression between karyotypes and sexes. Twenty‐six pathways were differentially expressed between αα and ββ (Table 3A). Of these, 10 were found in multiple tests. We found pathways related to cell cycle metabolism and control, such as nucleotide metabolism or amino acid metabolism as well as signaling (FoxO pathway) or genetic information processing (Fanconi anemia pathway). Twelve of the 26 pathways differing between karyotypes were also related to energetic metabolism, particularly in males, including fatty acid degradation, carbohydrate metabolism, and metabolism of cofactors. Of particular interest, male analysis included two organismal pathways, one related to longevity regulation and another involved in phototransduction in flies. As in other insects, increased size in *C. frigida* is associated with increased longevity and thus αα males live considerably longer on average (Butlin and Day 1985a). We found 16 pathways differentially expressed between males and females (Table 3B), including hormone biosynthesis.

**Table 3:** Functional pathways exhibiting subnetworks of genes interacting with each other and differentially expressed between karyotypes or sexes

A: Genotype effects								
Pathway	Category	Network size	Subnetwork size	Subnetwork score	*P*‐value	*Q*‐value	Analysis	Additional analyses where significant
Alanine, aspartate, and glutamate metabolism	Amino acid metabolism	26	6	3.7	0.019	0.162	Adult αα vs. ββ	Male αα vs. ββ
Glutathione metabolism	Amino acid metabolism	37	9	5.7	0.008	0.146	Female αα vs. ββ	Male αα vs. ββ
Arginine and proline metabolism	Amino acid metabolism	28	7	3.4	0.022	0.163	Male αα vs. ββ	
Phenylalanine metabolism	Amino acid metabolism	8	5	3.4	0.027	0.165	Adult αα vs. ββ	
Glycine, serine, and threonine metabolism	Amino acid metabolism	24	11	6.8	0.002	0.087	Female αα vs. ββ	Male αα vs. ββ, Adult αα vs. ββ
Thiamine metabolism	Amino acid metabolism	13	4	6.6	0.002	0.087	Female αα vs. ββ	Larvae αα vs. ββ
Tyrosine metabolism	Amino acid metabolism	17	5	3.1	0.035	0.173	Male αα vs. ββ	
Amino sugar and nucleotide sugar metabolism	Carbohydrate metabolism	38	4	6.0	0.011	0.193	Adult αα vs. ββ	
Glyoxylate and dicarboxylate metabolism	Carbohydrate metabolism	19	7	5.1	0.014	0.146	Female αα vs. ββ	Adult αα vs. ββ
Galactose metabolism	Carbohydrate metabolism	25	6	3.1	0.039	0.180	Male αα vs. ββ	
Starch and sucrose metabolism	Carbohydrate metabolism	27	8	3.7	0.008	0.083	Male αα vs. ββ	
Oxidative phosphorylation	Energy metabolism	32	5	4.1	0.004	0.057	Male αα vs. ββ	
Fanconi anemia pathway	Genetic information processing (replication and repair)	13	7	4.0	0.010	0.150	Adult αα vs. ββ	
Sphingolipid metabolism	Lipid metabolism	25	3	5.8	0.007	0.146	Female αα vs. ββ	
Ether lipid metabolism	Lipid metabolism	18	4	3.2	0.028	0.172	Male αα vs. ββ	
Fatty acid degradation	Lipid metabolism	28	18	6.8	0.001	0.060	Male αα vs. ββ	Adult αα vs. ββ
Fatty acid elongation	Lipid metabolism	14	4	7.0	0.001	0.060	Male αα vs. ββ	Adult αα vs. ββ
Glycerophospholipid metabolism	Lipid metabolism	49	6	4.1	0.004	0.057	Male αα vs. ββ	
One carbon pool by folate	Metabolism of cofactors and vitamins	11	6	5.2	0.014	0.146	Female αα vs. ββ	
Folate biosynthesis	Metabolism of cofactors and vitamins	29	5	4.2	0.003	0.057	Male αα vs. ββ	Adult αα vs. ββ, Female αα vs. ββ
Purine metabolism	Nucleotide metabolism	118	42	6.5	0.000	0.000	Adult αα vs. ββ	Male αα vs. ββ
Pyrimidine metabolism	Nucleotide metabolism	75	6	3.6	0.021	0.162	Adult αα vs. ββ	
Longevity regulating pathway—multiple species	Organismal system (aging)	41	3	3.5	0.023	0.162	Adult αα vs. ββ	Male αα vs. ββ
Phototransduction—fly	Organismal system (Sensory system)	26	6	4.0	0.005	0.057	Male αα vs. ββ	
FoxO signaling pathway	Signal transduction	46	6	3.3	0.031	0.176	Adult αα vs. ββ	
Neuroactive ligand‐receptor interaction	Signaling molecules	9	7	5.3	0.011	0.146	Female αα vs. ββ	

B: Sex effects								
Pathway	Category	Network Size	Subnetwork Size	Subnetwork Score	P‐value	Q‐value	Analysis	Additional analyses where significant
Alanine, aspartate, and glutamate metabolism	Amino acid metabolism	26	7	4.4	0.011	0.059	Sex	Adult αα vs. ββ, Male αα vs. ββ
Arginine and proline metabolism	Amino acid metabolism	28	4	6.8	0.000	0.000	Sex	Male αα vs. ββ
Drug metabolism—cytochrome P450	Xenobiotics biodegradation and metabolism	20	8	3.7	0.028	0.101	Sex	
Drug metabolism—other enzymes	Xenobiotics biodegradation and metabolism	33	5	3.6	0.033	0.110	Sex	
Folate biosynthesis	Metabolism of cofactors and vitamins	29	4	3.9	0.021	0.084	Sex	Adult αα vs. ββ, Female αα vs. ββ, Males αα vs. ββ
Galactose metabolism	Carbohydrate metabolism	25	7	3.8	0.022	0.084	Sex	Male αα vs. ββ
Glutathione metabolism	Amino acid metabolism	37	8	6.2	0.000	0.000	Sex	Male αα vs. ββ, Female αα vs. ββ
Glycerophospholipid metabolism	Lipid metabolism	49	7	3.4	0.046	0.140	Sex	Male αα vs. ββ
Glycolysis/gluconeogenesis	Carbohydrate metabolism	36	4	6.9	0.000	0.000	Sex	
Insect hormone biosynthesis	Metabolism of terpenoids and polyketides	24	8	3.5	0.038	0.113	Sex	
Longevity regulating pathway—multiple species	Organismal system (aging)	41	8	3.6	0.035	0.111	Sex	Adult αα vs. ββ, Male αα vs. ββ
Phototransduction—fly	Organismal system (Sensory system)	26	7	4.5	0.009	0.056	Sex	Male αα vs. ββ
Purine metabolism	Nucleotide metabolism	118	9	8.6	0.000	0.000	Sex	Adult αα vs. ββ, Male αα vs. ββ
Pyruvate metabolism	Carbohydrate metabolism	27	4	6.9	0.000	0.000	Sex	
Taurine and hypotaurine metabolism	Metabolism of other amino acids	9	3	4.0	0.017	0.083	Sex	
Valine, leucine, and isoleucine degradation	Amino acid metabolism	32	3	3.9	0.019	0.084	Sex	

For clarity, only karyotype effects are shown in panel A and sex effects are shown in panel B. Pathways are based on the KEGG database with genes identified in flybase. Significance of network score was assessed using the R library signet, by comparing with scores generated by random sampling. Network size is the number of genes connected in the pathways under consideration. Subnetworks are a subset of genes that are directly connected by edges and show high scoring. Subnetwork size is the number of genes and subnetwork score is the normalized score inferred by the procedure based on the strength of the relationship between the factor compared (karyotype/sex) and expression at the genes involved in this subnetwork. For A, if a term was significant in multiple analyses, we show the data from the most significant test and list that one in the analysis column. The additional tests are listed under “Additional analyses where significant”.

Taken together, these GO terms and the gene networks analysis reveal a clear and strong association with development and metabolism/energy storage, and cell cycle metabolism and genetic information processing, respectively. Overall, more terms for the effect of karyotype were associated with the male dataset compared to the female dataset (GO: two terms vs. one term, Signet: 15 pathways vs. eight pathways), although this is not surprising given the difference in the number of differentially expressed genes. These associations between inversion karyotype and metabolism and development are corroborated by the large phenotypic effects of *Cf‐Inv(1)*, which results in strong size and developmental time differences in males but not females (Butlin et al. 1982; Mérot et al. 2018).

There were fewer terms associated with the larvae. Overall, the signal in larvae was very weak and we only identified one pathway significantly differing between genotypes: thiamine metabolism, which is associated with digestion. This is not surprising as larvae stop feeding before pupation (Chown and Gaston 2010) and αα males develop 1.2–1.5× more slowly than ββ males. It should be noted that our larval samples were almost certainly in different stages of development as we standardized by time rather than stage. Work in *Drosophila melanogaster* shows that thiamine is critical for pupation (Sannino et al. 2018) further underlining that the differences we observe are likely partially linked to differences in developmental stage.

### COMBINING GENOMIC AND TRANSCRIPTOMIC STUDIES FACILITATES THE IDENTIFICATION OF CANDIDATE GENES

By combining our gene expression results with results from a previous study that identified environmentally associated SNP outliers (Mérot et al. 2021), we were also able to identify a small group of strong candidate genes for local adaptation. We compared the position of 997 transcripts that were differentially expressed between karyotypes in one of our six contrasts (adult αα vs. ββ, adult male αα vs. ββ, adult female αα vs. ββ, larvae αα vs. ββ, larvae αβ vs. ββ, larvae αα vs. αβ) with 1526 outlier SNPs identified as being associated with biotic and abiotic characteristics of the wrackbed, as these factors have been found to be significant selective forces on *Cf‐Inv(1)* (Day et al. 1983; Butlin and Day 1989; Mérot et al. 2018). We found 86 differentially expressed transcripts that mapped within 5 kb of an environmentally associated SNP. Randomly subsampling our tested transcripts 10,000 times indicated that the expected overlap should only be 42 ± 0.06 transcripts. This is likely due to the linkage disequilibrium created by the inversion, running this test using only transcripts that mapped to *Cf‐Inv(1)* generated an expectation closer to the observed value (expected overlap: 67 ± 0.06, actual: 70). Of our 86 overlapping transcripts, 55 were associated with one of two PCs that described seaweed composition of the wrackbed habitat, whereas 44 were associated with abiotic characteristics of the wrackbed such as depth, temperature, and salinity. There was some overlap; 13 transcripts were associated with both wrackbed composition and climate. All of the transcripts associated with abiotic characteristics were located within *Cf‐Inv(1)*. In contrast, 15 out of 55 transcripts associated with seaweed composition were located in other places in the genome. Full information on these loci can be found in Tables S5 and S6.

The wrackbed composition represents a major selective force both on *Cf‐Inv(1)* as well as on *C. frigida* as a whole. Flies raised on *Laminaria* spp. are larger and in better condition than flies raised on *Fucus* spp., although this effect is strongest in αα and αβ males (Edward 2008). These effects are likely tied directly to the microbial community of these algae, which forms the base of the *C. frigida* larval diet; *Fucus* spp. support large numbers of *Flavobacterium*, whereas *Pseudomonas* spp. are more common on *Laminaria* spp. (Laycock 1974; Bolinches et al. 1988). Thus, we expect some candidate genes to be related to either digestion or growth. Within our 55 candidates, we found several loci relating to digestive processes, such as carbonic anhydrase 5A that helps regulate pH of the midgut in *D. melanogaster* (Overend et al. 2016) and trypsin, a crucial digestive enzyme (Wu et al. 2009). As with the signet analysis, we also uncovered genes relating to the cessation of larval feeding and the onset of pupation, suggesting that the timing of this transition is a major factor underlying the size difference between αα and ββ males rather than differences in larval growth rate. In insects, two of the major modulators of feeding behavior are neuropeptide F (*npf*) and serotonin (5‐HT) (Fadda et al. 2019) (Neckameyer 2010). In older nonfeeding *Drosophila* larvae, *npf* is downregulated (Wu et al. 2003) and one potential mediator of this is tetrahydrobiopterin (BH4), a fat‐derived metabolite that suppresses the release of *npf* from *npf* neurons (Kim et al. 2017). Among our candidates was pterin‐4‐alpha‐carbinolamine dehydratase (*Pcd*) that is involved in the recycling of BH4 and thus increasing levels of BH4. In our data, *Pcd* was upregulated in ββ larvae and ββ males: this could suppress *npf* and thus feeding behavior leading to earlier pupation. 5‐HT is another major regulator of feeding behavior, and increased levels of 5‐HT in the gut of *D. melanogaster* enhance larval feeding behavior (Neckameyer 2010). Among our candidates was 5‐hydroxytryptamine receptor 1 (*HT1R*) that was upregulated in αα males, potentially increasing feeding behavior.

Abiotic characteristics are harder to associate with gene function than seaweed composition but we did find an abundance of genes involved in pupation, cuticle hardening, and eclosion such as *LGR5* and *LCR15* (Mendive et al. 2005), eclosion hormone (Krüger et al. 2015), and *ChT* (Hamid et al. 2019). Development time in *C. frigida* is highly plastic and is affected by temperature and density as well as karyotype (Butlin and Day 1984). As wrackbeds are ephemeral habitats, there is likely strong selection on these traits as well. Overall, these results provide some initial insights and putative candidates for further exploration. Furthermore, it is clear that many of the traits are likely polygenic and highly complex. Although merging transcriptomic and genomic datasets provides an excellent first step to narrow down candidates, more work, especially functional validation, needs to be done to differentiate between adaptive and linked variation.

## Conclusions

Abundant evidence indicates that chromosomal inversions are key genomic factors in eco‐evolutionary processes because of their multifarious impacts on genome structure, recombination, and regulation (Hoffmann and Rieseberg 2008; Wellenreuther and Bernatchez 2018). However, few studies have made progress toward dissecting the mechanistic pathways that enable inversions to shape evolutionary trajectories. Using a transcriptomic approach in the seaweed fly *Coelopa frigida* revealed that the impact of *Cf‐Inv(1)* was conditional and differed between males, females, and larvae. Males showed a stronger effect of *Cf‐Inv(1)* than females. Overall, most of the differentially expressed genes were *cis*‐regulated for karyotype, but not for sex effects. We found little evidence to implicate the breakpoints themselves or subsequent chromatin changes underlie these patterns, indicating that linked variation is likely the major cause of the observed expression differences. Interestingly, genes where the effect of *Cf‐Inv(1)* was more constant were more likely to be *cis*‐regulated than genes whose differential expression was conditional. These results suggest that *trans*‐regulation may be important for conditional gene expression in inversions. Combining our results with genomic data uncovered candidate variants in the inversion that may underlie mechanistic pathways that determine critical phenotypes in particular the cessation of larval feeding. Overall, our results highlight the complex effects of inversion polymorphisms on gene expression across contexts and the benefit of combining transcriptomic and genomic approaches in the study of inversions.

## Methods

### REARING AND CROSSES

Larvae of *C. frigida* for breeding were collected from the field in April/May 2017 from Skeie, Norway (58.69733, 5.54083), Østhassel, Norway (58.07068, 6.64346), Ystad, Sweden (55.425, 13.77254), and Smygehuk, Sweden (55.33715, 13.35963). Larvae were also collected from Skadbergsanden, Norway (58.45675, 5.91407) in June 2016. See Figure 1A for all sampling locations. All larvae were brought back live to the Tjärnö Marine Laboratory in Strömstad, Sweden where they were raised to adulthood at 25°C.

We generated an αα line from Skeie and a ββ line from each population (see the Supporting Information for details). Six days after the creation of these lines, two replicates of three larvae each from each line were flash frozen in liquid nitrogen and stored at −80°C until extraction. Larvae were always stored as groups of three henceforth referred to as larval pools. The adults that emerged from these lines were used to make subsequent crosses within and between karyotypes and populations to generate αβ and ββ larvae (see Table S7 for the crossing scheme). Adults were then flash frozen individually in liquid nitrogen and stored at −80°C until extraction. All experimental crosses were set up in a 50‐ml tube with a sponge for aeration and 4 g *Saccharina latissima* and 2 g *Fucus* spp. Six days after the creation of these crosses, one larval pool from each cross was flash frozen in liquid nitrogen and stored at −80°C until extraction. All larval pools and adults were processed at the same time of day (±1 h) to reduce variation. We were able to get larval pools from two successful crosses per cross type. We also generated an ontogeny series to ensure a comprehensive transcriptome (see Note in the Supporting Information).

### RNA EXTRACTION, LIBRARY PREPARATION, AND SEQUENCING

RNA from all samples was extracted following a TriZOL protocol (see Note in the Supporting Information). Only flies from our lab lines and crosses were sequenced: two larval pools per line (one αα and four ββ lines) and two larval pools from each subsequent cross type (see Table S5 for the crossing scheme). We also sequenced three Skeie αα adult males, three Skeie αα adult females, five Skeie ββ adult males, two Skeie ββ adult females, three Skadbergsanden ββ adult females, and one Ystad ββ adult female. We chose these samples to bias toward parents of the larval samples and endeavored to get a good distribution of genotypes. However, we were severely limited by RNA quality. All of these samples were submitted to SciLifeLab in Uppsala, Sweden for library preparation and sequencing. RNA was purified with Agencourt RNA clean XP before library preparation. Library preparation was done with the TruSeq stranded mRNA library preparation kit including polyA selection. Samples were sequenced on a NovaSeq S1 flowcell with 100 bp paired end reads (version 1 sequencing chemistry).

### TRANSCRIPTOME ASSEMBLY

We only used samples from the geographically close populations Skeie and Østhassel to construct our transcriptome to limit genetic variation between samples. Individual assemblies for two of the Skeie αα adult males, two of the Skeie αα adult females, two of the Skeie ββ adult males, two of the Skeie ββ adult females, both of the Østhassel ontogenetic pools spanning 0–348 h of development (as a single assembly), both of the Skeie αα larval pools (as a single assembly), and both of the Skeie ββ larval pools (as a single assembly) were done using Trinity version 2.9.1 (11 assemblies in total) (Haas et al. 2013). Prior to assembly, all reads were trimmed and adaptors removed using cutadapt 2.3 with Python 3.7.2 (Martin 2011). All assemblies were run through TransRate 1.0.1 (Smith‐Unna et al. 2016), a quality assessment tool for de novo transcriptomes that looks for artifacts, such as chimeras and incomplete assembly, and provides individual transcript and overall assembly scores. We retained all transcripts from each assembly classified by TransRate as “good.” These contigs were then merged using CD‐hit 4.8.1 (Fu et al. 2012) with a sequence identity threshold of 0.95, a word size of 10, and local sequence alignment coverage for the longer sequence at 0.005. Finally, the transcriptome was mapped to the genome assembly (Merot et al. 2020a) using GMAP 2018‐07‐04 (Wu and Watanabe 2005). The mapping coordinates for each transcript were extracted and in the event that two transcripts mapped to the same coordinates, only the longer transcript was retained. The mapping coordinates of all transcripts were retained for use in further analyses. The final transcriptome was annotated using the Trinotate pipeline with the Uniprot/Swiss‐Prot and Pfam databases (Downloaded on June 25, 2020) (Grabherr et al. 2011).

### DIFFERENTIAL EXPRESSION ANALYSIS

We used DESeq2 1.26.0 to determine which transcripts were differentially expressed between karyotypes and sexes (Love et al. 2014). The reads from all samples were trimmed and the adaptors were removed using cutadapt 2.3 with Python 3.7.2 (Martin 2011). The trimmed reads were then aligned to the reference transcriptome using bowtie2 2.3.5.1 (Langmead and Salzberg 2012) and quantified using RSEM (Li and Dewey 2011). The resulting genes.results files were prepared for use in DESeq2 using the Trinity script abundance_estimates_to_matrix.pl (Haas et al. 2013). These files were used as input for DESeq2 1.26.0 implemented in R (Love et al. 2014). Adults and larvae were analyzed separately and normalization was done by DESeq2. We removed all transcripts where the total count of reads (across all individuals) was less than 10. We also removed a single sample (Skeie ββ larvae pool 1) as hierarchical clustering using a distance matrix revealed that this sample was an extreme outlier. In DESeq2, our model for adults included both karyotype and sex and their interaction, whereas the model for larvae included karyotype and population. We did not include population in the adult model as 13 out of 17 samples came from the Skeie population. We further split adult males and females and analyzed them separately. Conventional thresholds (log2 fold change > 2, adjusted *P*‐value after correction for false discover rate < 5%) were used to identify differentially expressed transcripts. We tested for GO enrichment in our different sets of results using topGO (Alexa and Rahnenfuhrer 2010) with the elim algorithm and the Fisher's exact test implemented in R (Love et al. 2014). Manhattan distance matrices for all subgroups (males, females, and larvae) were calculated using the dist() function in R and PERMANOVA results were calculated using Adonis2 in the vegan package (Dixon 2003). Note that karyotype was always used at the first term as terms are added sequentially and models differed between subgroups.

### GENE SUBNETWORK ANALYSIS

To investigate the effect of inversion on expression in genes involved in common biological pathways, we performed a gene network analysis designed to detect polygenic selection using the R package *signet* (Gouy et al. 2017). This method defines subnetworks of genes that interact with each other, because they are known to be involved in the same biological pathway in the KEGG database, and present similar patterns attributed to selection; for example, covariation in expression levels. For this analysis, we used the *D. melanogaster* KEGG database and thus focused on the transcripts that matched a gene in Flybase (13,586 out of 26,239). Variation of expression levels between genotypes were analyzed in a multivariate framework with redundancy analysis (RDA), with and without sex as covariate, and scaled to a *z*‐score such that individual transcript scores have a mean of 0 and a standard deviation of 1 (following Rougeux et al. 2019). Following the recommendations of the *signet* procedure, each pathway of the KEGG database was parsed to score gene subnetworks using 10,000 iterations of simulated annealing. A null distribution of subnetwork scores was generated by random sampling to create 10,000 subnetworks of variable sizes. We pathways with a higher score than the null distribution as significant, that is, with a *P*‐value below 0.05, and a false discovery rate (*Q*‐value) of 0.20.

### OVERLAP WITH GENOMIC RESULTS

We combined our data with previously published population genomic data to identify loci that may contribute to local adaptation. Briefly, in our previous work, 16 populations of *C. frigida* were sampled along latitudinal and ecological gradients and sequenced at the whole‐genome level, and the association between SNPs and environmental variation was tested using a combination of two genotype‐environment association methods (LFMM2 and Baypass) (Merot et al. 2020a). Using our mapping coordinates, we identified transcripts located <5 kb from an outlier SNP defined by both of these association methods and differentially expressed between genotypes in at least one of our analyses.

### ALLELE‐SPECIFIC EXPRESSION

We used our set of αβ larvae to search for transcripts that showed ASE. RNA from each of our samples was mapped to our reference genome using bowtie2 2.3.5.1 (Langmead and Salzberg 2012). The alignment files were sorted and read groups were added using Picard 2.10.3 (http://broadinstitute.github.io/picard/). The resulting files were indexed with samtools (Li 2011) and SNPs were called using bcftools (Li 2011). We took the conservative approach of only examining loci that were fixed different between arrangements. SNPs were filtered by mean depth (>5), maximum percentage of missing samples (25%), and F_ST_ between α and β = 1, using vcftools (Danecek et al. 2011). We further retained only SNPs that had observations from at least three individuals. To test for ASE, we used the ASEP package (Fan et al. 2020) implemented in R (Love et al. 2014). This package uses multi‐individual information and accounts for multi‐SNP correlations within the transcripts. Using ASEP, we performed a one‐condition analysis to detect gene‐level ASE and corrected for multiple testing using the Benjamini and Hochberg (1995) method implemented in R with “p.adjust.” (Benjamini and Hochberg 1995). We considered contigs with an adjusted *P*‐value < 0.1 to be significant.

## AUTHOR CONTRIBUTIONS

ELB, RKB, KJ, and MW conceived the idea. ELB carried out the breeding experiments and lab work. ELB and CM analyzed the data. ELB and HP provided financing. ELB and MW wrote the manuscript with input from all authors.

## DATA ARCHIVING

Raw reads are available on the NCBI SRA (Bioproject PRJNA746238). Assembled transcriptome, annotation files, analysis scripts for the signet, differential expression, allele‐specific expression, and figures and the associated input files are all available on the Harvard Dataverse (https://dataverse.harvard.edu/dataverse/cfrigida_exp).

## Supporting information



Supplemental Figure 1 Differential expression by sex in adults.

Supplemental Table 3 ‐ Patterns of larval DE genes across contrasts.
Supplemental Table 4 ‐ Two‐sample two sided Kolmogorov‐Smirnov on distributions of log2fold values when comparing αα vs. ββ
Supplemental Table 5 ‐ Seaweed composition/Differential expression overlap.
Supplemental Table 6 ‐ Abiotic characters/Differential expression overlap.

Supplemental Table 1 ‐ Statistics on Individual Transcriptome Assemblies
Supplemental table 2 ‐ PERMANOVA results based on Manhattan distances.
Supplemental Table 3 ‐ Two‐sample two sided Kolmogorov‐Smirnov on distributions of log2fold values when comparing αα vs. ββ
Supplemental Table 4 ‐ Seaweed composition/Differential expression overlap.
Supplemental Table 5 ‐ Abiotic characters/Differential expression overlap.
Supplemental Table 6 ‐ Crossing scheme
