# Supplementary material for: A large chromosomal inversion shapes gene expression in seaweed flies (Coelopa frigida)
Source: Evol Lett. 2021 Oct 7;5(6):607–24. doi: 10.1002/evl3.260 (PMC8645196; doi:10.1002/evl3.260)
Supplement: Supplementary file 3 — Supplemental Table 1 ‐ Statistics on Individual Transcriptome Assemblies Supplemental table 2 ‐ PERMANOVA results based on Manhattan distances. Supplemental Table 3 ‐ Two‐sample two sided Kolmogorov‐Smirnov on distributions of log2fold values when comparing αα vs. ββ Supplemental Table 4 ‐ Seaweed composition/Differential expression overlap. Supplemental Table 5 ‐ Abiotic characters/Differential expression overlap. Supplemental Table 6 ‐ Crossing scheme [file EVL3-5-607-s001.pdf]

Supplemental Table 1 - Statistics on Individual Transcriptome Assemblies

Sample	Genotype	Sex	Life Stage	# Genes	# Transcripts	N50	Mean Length	Transrate Score	Proportion 'good' Transcripts*
P10M	αα	M	adult	26057	34924	1641	974	0.354	0.91
P1M	αα	M	adult	27373	36823	1979	1066	0.354	0.88
P13F	αα	F	adult	25537	37109	2680	1380	0.347	0.84
Pools	mixed	mixed	mixed	31237	40632	1447	863	0.299	0.95
SKAA pool	αα	mixed	larval	7880	9296	969	649	0.385	0.15
SKBB pool	ββ	mixed	larval	5700	6583	908	614	0.374	0.21
P21F	ββ	F	adult	23152	33057	2642	1378	0.35	0.80
P23F	ββ	F	adult	20155	25087	1717	1005	0.444	0.94
P23M	ββ	M	adult	32683	48558	2679	1357	0.331	0.83
P3F	αα	F	adult	28128	43277	2748	1436	0.359	0.89
P3M	ββ	M	adult	28518	36215	1348	813	0.336	0.93

* - as classified by Transrate

Supplemental table 2 - PERMANOVA results based on Manhattan distances. All terms possible are included in each model but karyotype is always the first term added sequentially

A. All adults Combined

Term	df	Sum of Squares	R2	F	P-value
Karyotype	1	374439873	0.08399	2.5483	0.049
Population	2	829377511	0.18605	2.8222	0.003
Sex	1	1490847756	0.33443	10.1461	0.001
Residual	12	1763248849	0.39553		
Total	16	4457913989	1		

B. Larvae

Term	df	Sum of Squares	R2	F	P-value
Karyotype	2	208651805	0.07286	1.0346	0.331
Population	4	739084448	0.2581	1.8325	0.057
Residual	19	1915821804	0.66904		
Total	25	2863558057	1		

C. Adult Males

Term	df	Sum of Squares	R2	F	P-value
Karyotype	1	287571012	0.2464	1.9618	0.096
Residual	6	879532921	0.7536		
Total	7	1167103933	1		

D. Adult Females

Term	df	Sum of Squares	R2	F	P-value
Karyotype	1	112267691	0.15348	1.3684	0.133
Population	2	209013786	0.28573	1.2738	0.148
Residual	5	410214221	0.56079		
Total	8	731495698	1		

Supplemental Table 3 - Two-sample two sided Kolmogorov-Smirnov on distributions of Log2Fold values when comparing αα vs. ββ

Group 1	Group 2	D value	P value
Female *Cf-Inv(1)*	Larvae *Cf-Inv(1)*	1.86E-01	< 2.2E-16
Female *Cf-Inv(1)*	Male *Cf-Inv(1)*	1.13E-01	< 2.2E-16
Female Global	Female *Cf-Inv(1)*	9.44E-02	< 2.2E-16
Male Global	Male *Cf-Inv(1)*	8.44E-02	< 2.2E-16
Larvae Global	Larvae *Cf-Inv(1)*	3.69E-02	1.64E-02

Supplemental Table 4 - Seaweed composition/Differential expression overlap. For log2 foldchange Positive values indicate higher expression in ββ and negative values indicate higher expression in αα. if a term was significant in multiple DE analyses we show the data from the most significant test and list that one in the analysis column. The additional tests are listed under 'Additional analyses where significant'.

Transcript	log2 FoldChange	Adjusted P-value	Analysis	Additional Analyses where significant	Environmental Association	Physical Location	sprot_Top_BLASTX_hit	sprot_Top_BLASTP_hit	uniprot_sprot.fasta_BLASTX	uniprot_sprot.fasta_BLASTP	Pfam	SignalP	TmHMM	eggnog	Kegg	gene_ontology_BLASTX	gene_ontology_BLASTP	gene_ontology_Pfam
P10M_DN24400_c0_g1_i1	-8.97	3.83E-16	Female αα vs. ββ	Adult αα vs. ββ, Male αα vs. ββ	Seaweed composition 2	*Cf-Inv(1)*	.	.	.	.	.	.	.	.	.	.	.	.
P10M_DN1168_c0_g1_i1	-8.77	2.33E-13	Male αα vs. ββ	Adult αα vs. ββ, Female αα vs. ββ	Seaweed composition 1&2, Abiotic characteristics	*Cf-Inv(1)*	.	.	.	.	.	.	.	.	.	.	.	.
P13F_DN370_c0_g3_i1	-7.34	4.14E-05	Male αα vs. ββ		Seaweed composition 1	*Cf-Inv(1)*	.	.	.	.	.	.	.	.	.	.	.	.
pool_DN31577_c0_g1_i1	-7.02	6.93E-06	Female αα vs. ββ	Adult αα vs. ββ	Seaweed composition 2, Abiotic characteristics	*Cf-Inv(1)*	.	.	.	.	.	.	.	.	.	.	.	.
P3F_DN5589_c0_g3_i1	-6.80	4.15E-07	Male αα vs. ββ	Adult αα vs. ββ, Female αα vs. ββ	Seaweed composition 2	*Cf-Inv(1)*	.	.	.	.	.	.	.	.	.	.	.	.
P13F_DN3531_c0_g1_i1	-6.76	6.44E-04	Male αα vs. ββ		Seaweed composition 2	*LG5*	FBCD1_MOUSE^FBCD1_MOUSE^Q:160-957,H:170-456^39.394%ID^E:2.83e-59^RecName: Full=Fibrinogen C domain-containing protein 1;^Eukaryota; Metazoa; Chordata; Craniata; Vertebrata; Euteleostomi; Mammalia; Eutheria; Euarchontoglires; Glires; Rodentia; Myomorpha; Muroidea; Muridae; Murinae; Mus; Mus	FBCD1_MOUSE^FBCD1_MOUSE^Q:54-319,H:170-456^39.394%ID^E:8.22e-60^RecName: Full=Fibrinogen C domain-containing protein 1;^Eukaryota; Metazoa; Chordata; Craniata; Vertebrata; Euteleostomi; Mammalia; Eutheria; Euarchontoglires; Glires; Rodentia; Myomorpha; Muroidea; Muridae; Murinae; Mus; Mus	60-957,H:170-456^39.3	-319,H:170-456^39.	amma chain	1^25^0.594	.	10ZYS4^fib	GG:mmu:98	nent^membrane`GO	nt^membrane`	.
P10M_DN15341_c0_g1_i1	-6.67	2.15E-07	Female αα vs. ββ	Adult αα vs. ββ, Male αα vs. ββ	Seaweed composition 2	*Cf-Inv(1)*	.	.	.	.	.	.	.	.	.	.	.	.
P1M_DN5313_c0_g1_i1	-6.44	2.64E-04	Male αα vs. ββ	Adult αα vs. ββ	Seaweed composition 2	*Cf-Inv(1)*	5HT1R_DROME^5HT1R_DROME^Q:3-212,H:495-564^98.571%ID^E:5.47e-40^RecName: Full=5-hydroxytryptamine receptor 1;^Eukaryota; Metazoa; Ecdysozoa; Arthropoda; Hexapoda; Insecta; Pterygota; Neoptera; Holometabola; Diptera; Brachycera; Muscomorpha; Ephydroidea; Drosophilidae; Drosophila; Sophophora	.	3-212,H:495-564^98.57	.	.	.	.	10XRW9^R	mel_CG1207	192^biological_proc	.	.
P13F_DN24828_c0_g1_i1	-5.91	1.45E-03	Adult αα vs. ββ	Female αα vs. ββ, Male αα vs. ββ	Seaweed composition 2	*Cf-Inv(1)*	.	.	.	.	.	.	.	.	.	.	.	.
pool_DN29225_c0_g1_i1	-5.89	2.99E-02	Female αα vs. ββ		Seaweed composition 1, Abiotic characteristics	*Cf-Inv(1)*	.	.	.	.	.	.	.	.	.	.	.	.
P10M_DN6612_c0_g1_i2	-5.47	1.84E-04	Male αα vs. ββ		Seaweed composition 2	*Cf-Inv(1)*	.	.	.	.	.	.	.	.	.	.	.	.
P10M_DN14069_c0_g1_i1	-4.89	1.22E-02	Male αα vs. ββ		Seaweed composition 2	*LG5*	.	IR21A_DROME^IR21A_DROME^Q:171-376,H:472-698^22.034%ID^E:4.64e-06^RecName: Full=Ionotropic receptor 21a {ECO:0000312|EMBL:AAF51569.2};^Eukaryota; Metazoa; Ecdysozoa; Arthropoda; Hexapoda; Insecta; Pterygota; Neoptera; Holometabola; Diptera; Brachycera; Muscomorpha; Ephydroidea; Drosophilidae; Drosophila; Sophophora	2-1129,H:472-698^22.8	-376,H:472-698^22.	nd-gated ion	.	ology=o70-9	^Ligand-gat	dme:Dmel_C	.	ligand-gated io	nate receptor activity`GO:0016020^cellular_component^membrane
P10M_DN25899_c0_g1_i1	-4.02	2.26E-02	Male αα vs. ββ		Seaweed composition 1	*LG1*	.	.	.	.	.	.	.	.	.	.	.	.
P1M_DN3480_c0_g1_i1	-4.00	3.72E-10	Male αα vs. ββ		Seaweed composition 1	*Cf-Inv(1)*	CHIT_MANSE^CHIT_MANSE^Q:1536-2759,H:19-427^73.35%ID^E:0^RecName: Full=Endochitinase;^Eukaryota; Metazoa; Ecdysozoa; Arthropoda; Hexapoda; Insecta; Pterygota; Neoptera; Holometabola; Lepidoptera; Glossata; Ditrysia; Bombycoidea; Sphingidae; Sphinginae; Sphingini; Manduca`CHIT_MANSE^CHIT_MANSE^Q:3018-3188,H:496-553^46.552%ID^E:1.76e-08^RecName: Full=Endochitinase;^Eukaryota; Metazoa; Ecdysozoa; Arthropoda; Hexapoda; Insecta; Pterygota; Neoptera; Holometabola; Lepidoptera; Glossata; Ditrysia; Bombycoidea; Sphingidae; Sphinginae; Sphingini; Manduca	CHIT_MANSE^CHIT_MANSE^Q:23-573,H:19-553^62.929%ID^E:0^RecName: Full=Endochitinase;^Eukaryota; Metazoa; Ecdysozoa; Arthropoda; Hexapoda; Insecta; Pterygota; Neoptera; Holometabola; Lepidoptera; Glossata; Ditrysia; Bombycoidea; Sphingidae; Sphinginae; Sphingini; Manduca	E:0^.^.`P36362^P36362	Q:23-573,H:19-553^	8e-92`PF016	1^21^0.837	.	.	.	unction^chitinase a	ction^chitinase	n binding`GO:0006030^biological_process^chitin metabolic process`GO:0005576
P10M_DN29_c0_g1_i15	-3.94	1.51E-08	Male αα vs. ββ	Adult αα vs. ββ, Female αα vs. ββ	Seaweed composition 1	*Cf-Inv(1)*	.	TRYP_ASTAS^TRYP_ASTAS^Q:21-164,H:95-235^44.295%ID^E:4.5e-27^RecName: Full=Trypsin-1;^Eukaryota; Metazoa; Ecdysozoa; Arthropoda; Crustacea; Multicrustacea; Malacostraca; Eumalacostraca; Eucarida; Decapoda; Pleocyemata; Astacidea; Astacoidea; Astacidae; Astacus	.95e-28^.^.`Q2KJ63^Q2	1-164,H:95-235^44.2	sin^Trypsin^	.	.	.	.	.	ding`GO:00042	dopeptidase activity`GO:0006508^biological_process^proteolysis
P3F_DN3092_c0_g2_i4	-3.85	5.17E-03	Male αα vs. ββ	Adult αα vs. ββ, Female αα vs. ββ	Seaweed composition 2	*Cf-Inv(1)*	MARH5_MOUSE^MARH5_MOUSE^Q:631-1503,H:11-276^52.703%ID^E:6.52e-97^RecName: Full=E3 ubiquitin-protein ligase MARCHF5;^Eukaryota; Metazoa; Chordata; Craniata; Vertebrata; Euteleostomi; Mammalia; Eutheria; Euarchontoglires; Glires; Rodentia; Myomorpha; Muroidea; Muridae; Murinae; Mus; Mus	MARH5_MOUSE^MARH5_MOUSE^Q:75-188,H:11-124^63.158%ID^E:5.29e-53^RecName: Full=E3 ubiquitin-protein ligase MARCHF5;^Eukaryota; Metazoa; Chordata; Craniata; Vertebrata; Euteleostomi; Mammalia; Eutheria; Euarchontoglires; Glires; Rodentia; Myomorpha; Muroidea; Muridae; Murinae; Mus; Mus	631-1503,H:11-276^52.	.	G-variant do	.	.	ane-associat	nu:69104`K	O:0008270^molecu	0008270^mole	lecular_function^zinc ion binding
P10M_DN4148_c0_g1_i3	-3.73	4.00E-03	Female αα vs. ββ	Adult αα vs. ββ	Seaweed composition 2	*Cf-Inv(1)*	.	.	.	.	unknown fu	.	.	.	.	.	.	.

P1M_DN743_c0_g1_i1	-3.42	2.15E-04	Male αα vs. ββ		Seaweed composition 2	*LG4*	MYRO1_BREBR^MYRO1_BREBR^Q:162-1532,H:5-461^45.14%ID^E:2.62e-136^RecName: Full=Myrosinase 1 {ECO:0000303|PubMed:11804799};^Eukaryota; Metazoa; Ecdysozoa; Arthropoda; Hexapoda; Insecta; Pterygota; Neoptera; Paraneoptera; Hemiptera; Sternorrhyncha; Aphidomorpha; Aphidoidea; Aphididae; Macrosiphini; Brevicoryne	MYRO1_BREBR^MYRO1_BREBR^Q:43-499,H:5-461^45.14%ID^E:2.97e-138^RecName: Full=Myrosinase 1 {ECO:0000303|PubMed:11804799};^Eukaryota; Metazoa; Ecdysozoa; Arthropoda; Hexapoda; Insecta; Pterygota; Neoptera; Paraneoptera; Hemiptera; Sternorrhyncha; Aphidomorpha; Aphidoidea; Aphididae; Macrosiphini; Brevicoryne	62-1532,H:5-461^45.14	3-499,H:5-461^45.1	cosyl hydrol	1^20^0.747	.	.	.	n binding`GO:00191	binding`GO:001	lycosyl compounds`GO:0005975^biological_process^carbohydrate metabolic pr
P1M_DN2711_c0_g1_i2	-3.11	1.22E-06	Male αα vs. ββ	Adult αα vs. ββ, Female αα vs. ββ	Seaweed composition 2, Abiotic characteristics	*Cf-Inv(1)*	P5CR1_PONAB^P5CR1_PONAB^Q:945-1322,H:113-238^53.968%ID^E:1.28e-40^RecName: Full=Pyrroline-5-carboxylate reductase 1, mitochondrial;^Eukaryota; Metazoa; Chordata; Craniata; Vertebrata; Euteleostomi; Mammalia; Eutheria; Euarchontoglires; Primates; Haplorrhini; Catarrhini; Hominidae; Pongo`P5CR1_PONAB^P5CR1_PONAB^Q:590-871,H:12-106^41.053%ID^E:1.28e-40^RecName: Full=Pyrroline-5-carboxylate reductase 1, mitochondrial;^Eukaryota; Metazoa; Chordata; Craniata; Vertebrata; Euteleostomi; Mammalia; Eutheria; Euarchontoglires; Primates; Haplorrhini; Catarrhini; Hominidae; Pongo	P5CR2_BOVIN^P5CR2_BOVIN^Q:1-148,H:121-268^50%ID^E:1.72e-47^RecName: Full=Pyrroline-5-carboxylate reductase 2;^Eukaryota; Metazoa; Chordata; Craniata; Vertebrata; Euteleostomi; Mammalia; Eutheria; Laurasiatheria; Artiodactyla; Ruminantia; Pecora; Bovidae; Bovinae; Bos	:1.28e-40^.^.`Q5R9X6^	1-148,H:121-268^50	rboxylate re	.	.	-5-carboxyla	a:504987`KO	process^cellular re	ocess^cellular	.
P3F_DN12206_c0_g1_i1	-2.83	5.85E-04	Female αα vs. ββ	Adult αα vs. ββ, Male αα vs. ββ	Seaweed composition 2, Abiotic characteristics	*Cf-Inv(1)*	.	.	.	.	^Peptidase	1^28^0.772	.	.	.	.	.	biological_process^proteolysis
SKBB_DN2192_c0_g1_i1	-2.51	3.05E-02	Male αα vs. ββ		Seaweed composition 2, Abiotic characteristics	*Cf-Inv(1)*	.	.	.	.	uperoxide dis	.	.	.	.	.	.	ng`GO:0006801^biological_process^superoxide metabolic process
P10M_DN2169_c0_g1_i2	-2.47	2.74E-04	Male αα vs. ββ		Seaweed composition 1, Abiotic characteristics	*Cf-Inv(1)*	.	.	.	.	.	.	Hel=2^Topo	.	.	.	.	.
P1M_DN646_c0_g3_i1	-2.42	2.18E-02	Male αα vs. ββ		Seaweed composition 1	*LG3*	C4D20_DROME^C4D20_DROME^Q:319-1842,H:3-506^54.921%ID^E:0^RecName: Full=Probable cytochrome P450 4d20;^Eukaryota; Metazoa; Ecdysozoa; Arthropoda; Hexapoda; Insecta; Pterygota; Neoptera; Holometabola; Diptera; Brachycera; Muscomorpha; Ephydroidea; Drosophilidae; Drosophila; Sophophora	C4D20_DROME^C4D20_DROME^Q:4-511,H:3-506^54.921%ID^E:0^RecName: Full=Probable cytochrome P450 4d20;^Eukaryota; Metazoa; Ecdysozoa; Arthropoda; Hexapoda; Insecta; Pterygota; Neoptera; Holometabola; Diptera; Brachycera; Muscomorpha; Ephydroidea; Drosophilidae; Drosophila; Sophophora	^Q:319-1842,H:3-506^5	^Q:4-511,H:3-506^5	ochrome P4	1^17^0.786	.	4^Cytochrom	mel_CG1676	:0004497^molecula	004497^molec	ncorporation or reduction of molecular oxygen`GO:0020037^molecular_function
P10M_DN3574_c0_g2_i1	-2.29	4.23E-03	Male αα vs. ββ		Seaweed composition 2	*Cf-Inv(1)*	.	.	.	.	.	.	.	.	.	.	.	.
P3F_DN20033_c0_g1_i1	-2.25	1.92E-03	Male αα vs. ββ		Seaweed composition 1	*Cf-Inv(1)*	EST1_CULPI^EST1_CULPI^Q:307-1887,H:13-539^36.617%ID^E:1.81e-119^RecName: Full=Esterase B1;^Eukaryota; Metazoa; Ecdysozoa; Arthropoda; Hexapoda; Insecta; Pterygota; Neoptera; Holometabola; Diptera; Nematocera; Culicoidea; Culicidae; Culicinae; Culicini; Culex; Culex	EST1_CULPI^EST1_CULPI^Q:40-566,H:13-539^36.617%ID^E:6.51e-122^RecName: Full=Esterase B1;^Eukaryota; Metazoa; Ecdysozoa; Arthropoda; Hexapoda; Insecta; Pterygota; Neoptera; Holometabola; Diptera; Nematocera; Culicoidea; Culicidae; Culicinae; Culicini; Culex; Culex	7-1887,H:13-539^36.61	.	124`PF0785	.	.	.	.	nction^methyl indol	ion^methyl in	ecular_function^hydrolase activity
P3F_DN255_c0_g1_i2	-2.23	8.57E-06	Male αα vs. ββ		Seaweed composition 2	*Cf-Inv(1)*	GPDA_DROME^GPDA_DROME^Q:528-1577,H:1-350^91.714%ID^E:0^RecName: Full=Glycerol-3-phosphate dehydrogenase [NAD(+)], cytoplasmic;^Eukaryota; Metazoa; Ecdysozoa; Arthropoda; Hexapoda; Insecta; Pterygota; Neoptera; Holometabola; Diptera; Brachycera; Muscomorpha; Ephydroidea; Drosophilidae; Drosophila; Sophophora	GPDA_DROME^GPDA_DROME^Q:1-350,H:1-350^91.714%ID^E:0^RecName: Full=Glycerol-3-phosphate dehydrogenase [NAD(+)], cytoplasmic;^Eukaryota; Metazoa; Ecdysozoa; Arthropoda; Hexapoda; Insecta; Pterygota; Neoptera; Holometabola; Diptera; Brachycera; Muscomorpha; Ephydroidea; Drosophilidae; Drosophila; Sophophora	Q:528-1577,H:1-350^9	.	e-54`PF074	.	.	l-3-phospha	mel_CG9042	s^ethanol metaboli	ethanol metab	nate catabolic process`GO:0055114^biological_process^oxidation-reduction proc
P1M_DN453_c0_g1_i1	-2.19	1.15E-02	Female αα vs. ββ		Seaweed composition 2	*Cf-Inv(1)*	.	.	.	.	.	:1^20^0.66^	.	.	.	.	.	.
pool_DN211_c0_g1_i4	-2.12	1.35E-02	Male αα vs. ββ		Seaweed composition 2	*Cf-Inv(1)*	MLR_DROME^MLR_DROME^Q:257-694,H:68-213^95.89%ID^E:8.64e-87^RecName: Full=Myosin regulatory light chain 2;^Eukaryota; Metazoa; Ecdysozoa; Arthropoda; Hexapoda; Insecta; Pterygota; Neoptera; Holometabola; Diptera; Brachycera; Muscomorpha; Ephydroidea; Drosophilidae; Drosophila; Sophophora	MLR_DROME^MLR_DROME^Q:45-212,H:46-213^95.833%ID^E:6.87e-113^RecName: Full=Myosin regulatory light chain 2;^Eukaryota; Metazoa; Ecdysozoa; Arthropoda; Hexapoda; Insecta; Pterygota; Neoptera; Holometabola; Diptera; Brachycera; Muscomorpha; Ephydroidea; Drosophilidae; Drosophila; Sophophora	257-694,H:68-213^95.8	.	^E:3.7e-05`P	.	.	Calcium-bind	dme:Dmel_C	on binding`GO:0060	binding`GO:00	cular_function^calcium ion binding

P10M_DN7180_c0_g1_i1	-2.04	6.53E-03	Male αα vs. ββ		Seaweed composition 2	*LG5*	.	AMACR_RAT^AMACR_RAT^Q:1-377,H:1-382^50.785%ID^E:1.77e-133^RecName: Full=Alpha-methylacyl-CoA racemase;^Eukaryota; Metazoa; Chordata; Craniata; Vertebrata; Euteleostomi; Mammalia; Eutheria; Euarchontoglires; Glires; Rodentia; Myomorpha; Muroidea; Muridae; Murinae; Rattus	91-1421,H:1-382^50.78	-377,H:1-382^50.785	A-transfera	.	.	ydratase bil	.	.	A-transferase	lecular_function^catalytic activity
P23M_DN766_c0_g1_i1	2.06	4.04E-03	Male αα vs. ββ		Seaweed composition 2	*Cf-Inv(4.1)*	AG5L1_CULNU^AG5L1_CULNU^Q:81-686,H:2-208^44.34%ID^E:7.37e-51^RecName: Full=Antigen 5 like allergen Cul n 1 {ECO:0000303|PubMed:20537727};^Eukaryota; Metazoa; Ecdysozoa; Arthropoda; Hexapoda; Insecta; Pterygota; Neoptera; Holometabola; Diptera; Nematocera; Chironomoidea; Ceratopogonidae; Ceratopogoninae; Culicoides; Monoculicoides	AG5L1_CULNU^AG5L1_CULNU^Q:1-201,H:2-208^43.128%ID^E:3.61e-51^RecName: Full=Antigen 5 like allergen Cul n 1 {ECO:0000303|PubMed:20537727};^Eukaryota; Metazoa; Ecdysozoa; Arthropoda; Hexapoda; Insecta; Pterygota; Neoptera; Holometabola; Diptera; Nematocera; Chironomoidea; Ceratopogonidae; Ceratopogoninae; Culicoides; Monoculicoides	:81-686,H:2-208^44.34	-201,H:2-208^43.12	secretory p	1^18^0.928	.	.	.	lular_component^ex	ar_component^	.
P23M_DN5718_c0_g1_i1	2.14	4.03E-03	Male αα vs. ββ		Seaweed composition 1	*LG4*	.	.	.	.	.	.	.	.	.	.	.	.
P21F_DN1748_c0_g1_i3	2.17	3.32E-03	Male αα vs. ββ		Seaweed composition 1, Abiotic characteristics	*Cf-Inv(1)*	FACD2_HUMAN^FACD2_HUMAN^Q:412-4401,H:45-1399^23.883%ID^E:3.57e-76^RecName: Full=Fanconi anemia group D2 protein;^Eukaryota; Metazoa; Chordata; Craniata; Vertebrata; Euteleostomi; Mammalia; Eutheria; Euarchontoglires; Primates; Haplorrhini; Catarrhini; Hominidae; Homo	FACD2_HUMAN^FACD2_HUMAN^Q:109-1465,H:45-1438^24.096%ID^E:2.04e-85^RecName: Full=Fanconi anemia group D2 protein;^Eukaryota; Metazoa; Chordata; Craniata; Vertebrata; Euteleostomi; Mammalia; Eutheria; Euarchontoglires; Primates; Haplorrhini; Catarrhini; Hominidae; Homo	12-4401,H:45-1399^23	9-1465,H:45-1438^2	a protein Fa	.	.	i anemia co	sa:2177`KO:	meiotic recombinati	iotic recombin	biological_process^DNA repair
P3M_DN3450_c0_g1_i1	2.84	2.26E-02	Male αα vs. ββ		Seaweed composition 1	*LG4*	.	.	.	.	.	1^15^0.638	.	.	.	.	.	.
pool_DN8843_c0_g2_i1	2.99	4.70E-02	Male αα vs. ββ		Seaweed composition 1	*LG3*	.	.	.	.	.	.	.	.	.	.	.	.
P23M_DN467_c0_g6_i1	3.47	4.96E-07	Male αα vs. ββ	Adult αα vs. ββ, Female αα vs. ββ	Seaweed composition 1	*Cf-Inv(1)*	.	.	.	.	.	.	.	.	.	.	.	.
P23M_DN15156_c0_g1_i1	3.83	2.87E-02	Male αα vs. ββ		Seaweed composition 2	*Cf-Inv(1)*	.	.	.	.	.	.	.	.	.	.	.	.
P23M_DN1785_c0_g1_i2	3.86	2.38E-02	Larvae αα vs. ββ	Male αα vs. ββ	Seaweed composition 1&2	*Cf-Inv(1)*	PHS_DROVI^PHS_DROVI^Q:521-805,H:7-101^78.947%ID^E:1.47e-52^RecName: Full=Pterin-4-alpha-carbinolamine dehydratase;^Eukaryota; Metazoa; Ecdysozoa; Arthropoda; Hexapoda; Insecta; Pterygota; Neoptera; Holometabola; Diptera; Brachycera; Muscomorpha; Ephydroidea; Drosophilidae; Drosophila	PHS_DROVI^PHS_DROVI^Q:69-168,H:2-101^79%ID^E:8.18e-58^RecName: Full=Pterin-4-alpha-carbinolamine dehydratase;^Eukaryota; Metazoa; Ecdysozoa; Arthropoda; Hexapoda; Insecta; Pterygota; Neoptera; Holometabola; Diptera; Brachycera; Muscomorpha; Ephydroidea; Drosophilidae; Drosophila	521-805,H:7-101^78.94	.	a carbinola	.	.	etrahydrobi	.	ehydratase activity`G	dratase activit	ydratase activity`GO:0006729^biological_process^tetrahydrobiopterin biosynth
P23M_DN1100_c0_g4_i1	3.92	5.16E-06	Male αα vs. ββ	Adult αα vs. ββ, Female αα vs. ββ	Seaweed composition 1, Abiotic characteristics	*Cf-Inv(1)*	ZG49_XENLA^ZG49_XENLA^Q:394-594,H:62-128^43.284%ID^E:6.15e-11^RecName: Full=Gastrula zinc finger protein XlCGF49.1;^Eukaryota; Metazoa; Chordata; Craniata; Vertebrata; Euteleostomi; Amphibia; Batrachia; Anura; Pipoidea; Pipidae; Xenopodinae; Xenopus; Xenopus`ZG49_XENLA^ZG49_XENLA^Q:388-591,H:32-99^35.294%ID^E:8.08e-08^RecName: Full=Gastrula zinc finger protein XlCGF49.1;^Eukaryota; Metazoa; Chordata; Craniata; Vertebrata; Euteleostomi; Amphibia; Batrachia; Anura; Pipoidea; Pipidae; Xenopodinae; Xenopus; Xenopus`ZG49_XENLA^ZG49_XENLA^Q:388-534,H:88-136^48.98%ID^E:4.04e- [illegible]	ZN484_HUMAN^ZN484_HUMAN^Q:130-285,H:382-518^31.21%ID^E:3.62e-14^RecName: Full=Zinc finger protein 484;^Eukaryota; Metazoa; Chordata; Craniata; Vertebrata; Euteleostomi; Mammalia; Eutheria; Euarchontoglires; Primates; Haplorrhini; Catarrhini; Hominidae; Homo`ZN484_HUMAN^ZN484_HUMAN^Q:130-211,H:746-827^43.902%ID^E:4.53e-12^RecName: Full=Zinc finger protein 484;^Eukaryota; Metazoa; Chordata; Craniata; Vertebrata; Euteleostomi; Mammalia; Eutheria; Euarchontoglires; Primates; Haplorrhini; Catarrhini; Hominidae; Homo`ZN484_HUMAN^ZN484_HUMAN^Q:92-197,H:662-757^35.849%ID^E:1e-10^RecName: Full=Zinc finger protein 484;^Eukaryota; Metazoa; Chordata; Craniata; [illegible]	:8.08e-08^.^.`P18724^P	.	nger, C2H2 t	.	.	8^Zinc finge	a:83744`KO	ecular_function^DN	2^molecular_fu	cular_function^nucleic acid binding
P21F_DN12134_c0_g1_i1	4.02	2.00E-02	Female αα vs. ββ		Seaweed composition 2	*Cf-Inv(4.1)*	LEDL2_DROME^LEDL2_DROME^Q:147-545,H:35-172^34.752%ID^E:1.52e-16^RecName: Full=C-type lectin 37Da {ECO:0000305};^Eukaryota; Metazoa; Ecdysozoa; Arthropoda; Hexapoda; Insecta; Pterygota; Neoptera; Holometabola; Diptera; Brachycera; Muscomorpha; Ephydroidea; Drosophilidae; Drosophila; Sophophora	LEDL2_DROME^LEDL2_DROME^Q:32-164,H:35-172^34.752%ID^E:6.28e-17^RecName: Full=C-type lectin 37Da {ECO:0000305};^Eukaryota; Metazoa; Ecdysozoa; Arthropoda; Hexapoda; Insecta; Pterygota; Neoptera; Holometabola; Diptera; Brachycera; Muscomorpha; Ephydroidea; Drosophilidae; Drosophila; Sophophora	147-545,H:35-172^34.7	2-164,H:35-172^34.7	ctin C-type d	1^18^0.641	.	X0^Lectin C-	me:Dmel_C	lar_function^metal	r_function^met	.
P10M_DN609_c0_g1_i1	4.47	3.44E-06	Male αα vs. ββ		Seaweed composition 2	*LG5*	.	.	.	.	.	.	.	.	.	.	.	.
P23M_DN1261_c0_g1_i1	4.84	3.91E-05	Male αα vs. ββ	Larvae αα vs. ββ	Seaweed composition 1	*LG3*	.	.	.	.	nknown fun	1^19^0.915	.	.	.	.	.	.

P23F_DN19286_c0_g1_i1	4.88	2.09E-03	Adult αα vs. ββ	Female αα vs. ββ	Seaweed composition 2	*Cf-Inv(1)*	ZG26_XENLA^ZG26_XENLA^Q:136-453,H:144-253^31.818%ID^E:4.55e-12^RecName: Full=Gastrula zinc finger protein XlCGF26.1;^Eukaryota; Metazoa; Chordata; Craniata; Vertebrata; Euteleostomi; Amphibia; Batrachia; Anura; Pipoidea; Pipidae; Xenopodinae; Xenopus; Xenopus`ZG26_XENLA^ZG26_XENLA^Q:97-450,H:215-337^33.333%ID^E:1.85e-11^RecName: Full=Gastrula zinc finger protein XlCGF26.1;^Eukaryota; Metazoa; Chordata; Craniata; Vertebrata; Euteleostomi; Amphibia; Batrachia; Anura; Pipoidea; Pipidae; Xenopodinae; Xenopus; Xenopus`ZG26_XENLA^ZG26_XENLA^Q:136-453,H:88-169^28.302%ID^E:1.32e-10^RecName: Full=Gastrula	ZG26_XENLA^ZG26_XENLA^Q:46-151,H:144-253^32.727%ID^E:6.75e-14^RecName: Full=Gastrula zinc finger protein XlCGF26.1;^Eukaryota; Metazoa; Chordata; Craniata; Vertebrata; Euteleostomi; Amphibia; Batrachia; Anura; Pipoidea; Pipidae; Xenopodinae; Xenopus; Xenopus`ZG26_XENLA^ZG26_XENLA^Q:33-150,H:215-337^33.333%ID^E:2.73e-13^RecName: Full=Gastrula zinc finger protein XlCGF26.1;^Eukaryota; Metazoa; Chordata; Craniata; Vertebrata; Euteleostomi; Amphibia; Batrachia; Anura; Pipoidea; Pipidae; Xenopodinae; Xenopus; Xenopus`ZG26_XENLA^ZG26_XENLA^Q:46-151,H:88-197^31.818%ID^E:1.33e-11^RecName: Full=Gastrula zinc finger protein XlCGF26.1;^Eukaryota; Metazoa; Chordata; Craniata; Vertebrata; Euteleostomi; Amphibia;	E:1.32e-10^.^.`P18715^	Q:46-151,H:88-197^	c finger, C2H	.	.	.	.	ecular_function^DNA	lar_function^D	cular_function^nucleic acid binding
P21F_DN2673_c0_g2_i1	5.31	1.15E-09	Female αα vs. ββ	Adult αα vs. ββ, Male αα vs. ββ	Seaweed composition 2	*Cf-Inv(1)*	.	.	.	.	.	.	.	.	.	.	.	.
P23M_DN2932_c0_g2_i1	5.55	2.21E-03	Adult αα vs. ββ	Female αα vs. ββ, Male αα vs. ββ	Seaweed composition 2	*Cf-Inv(1)*	.	.	.	.	.	.	.	.	.	.	.	.
P3M_DN3188_c0_g1_i3	5.83	3.32E-02	Male αα vs. ββ		Seaweed composition 1, Abiotic characteristics	*Cf-Inv(1)*	DOCK9_HUMAN^DOCK9_HUMAN^Q:494-604,H:14-50^62.162%ID^E:2.55e-06^RecName: Full=Dedicator of cytokinesis protein 9 {ECO:0000305};^Eukaryota; Metazoa; Chordata; Craniata; Vertebrata; Euteleostomi; Mammalia; Eutheria; Euarchontoglires; Primates; Haplorrhini; Catarrhini; Hominidae; Homo	.	494-604,H:14-50^62.16	.	.	.	.	Y^Dedicator	a:23348`KO	ction^guanyl-nucleo	.	.
P23M_DN5126_c0_g3_i1	6.17	7.80E-04	Female αα vs. ββ	Adult αα vs. ββ	Seaweed composition 2	*Cf-Inv(1)*	.	.	.	.	.	.	.	.	.	.	.	.
P23M_DN24621_c0_g1_i1	6.20	1.71E-03	Female αα vs. ββ	Adult αα vs. ββ, Male αα vs. ββ	Seaweed composition 2, Abiotic characteristics	*Cf-Inv(1)*	SYMM_DROME^SYMM_DROME^Q:1-249,H:466-556^49.451%ID^E:3.28e-20^RecName: Full=Methionine--tRNA ligase, mitochondrial {ECO:0000303|PubMed:22448145, ECO:0000312|FlyBase:FBgn0027083};^Eukaryota; Metazoa; Ecdysozoa; Arthropoda; Hexapoda; Insecta; Pterygota; Neoptera; Holometabola; Diptera; Brachycera; Muscomorpha; Ephydroidea; Drosophilidae; Drosophila; Sophophora	SYMM_DROME^SYMM_DROME^Q:1-100,H:466-566^45.37%ID^E:2.02e-20^RecName: Full=Methionine--tRNA ligase, mitochondrial {ECO:0000303|PubMed:22448145, ECO:0000312|FlyBase:FBgn0027083};^Eukaryota; Metazoa; Ecdysozoa; Arthropoda; Hexapoda; Insecta; Pterygota; Neoptera; Holometabola; Diptera; Brachycera; Muscomorpha; Ephydroidea; Drosophilidae; Drosophila; Sophophora	1-249,H:466-556^49.45	-100,H:466-566^45.3	.	.	.	initiation of	mel_CG3132	unction^ATP binding	tion^ATP bind	.
P23M_DN1251_c0_g1_i4	6.41	3.87E-04	Adult αα vs. ββ	Female αα vs. ββ, Male αα vs. ββ	Seaweed composition 2	*Cf-Inv(1)*	CAH5A_HUMAN^CAH5A_HUMAN^Q:179-3,H:102-160^44.068%ID^E:2.21e-07^RecName: Full=Carbonic anhydrase 5A, mitochondrial;^Eukaryota; Metazoa; Chordata; Craniata; Vertebrata; Euteleostomi; Mammalia; Eutheria; Euarchontoglires; Primates; Haplorrhini; Catarrhini; Hominidae; Homo	CAH4_BOVIN^CAH4_BOVIN^Q:111-157,H:97-143^51.064%ID^E:4.04e-09^RecName: Full=Carbonic anhydrase 4;^Eukaryota; Metazoa; Chordata; Craniata; Vertebrata; Euteleostomi; Mammalia; Eutheria; Laurasiatheria; Artiodactyla; Ruminantia; Pecora; Bovidae; Bovinae; Bos	179-3,H:102-160^44.06	1-157,H:97-143^51.0	yotic-type ca	.	.	^Carbonic a	a:280741`KO	ar_function^carbona	membrane`GO:	.
P23M_DN114_c0_g1_i1	7.19	2.84E-05	Female αα vs. ββ	Adult αα vs. ββ, Male αα vs. ββ	Seaweed composition 2	*Cf-Inv(1)*	.	.	.	.	nknown fun	.	.	.	.	.	.	.
P23M_DN1273_c0_g2_i1	7.96	3.50E-07	Male αα vs. ββ	Adult αα vs. ββ, Female αα vs. ββ	Seaweed composition 1&2	*Cf-Inv(1)*	.	.	.	.	.	.	.	.	.	.	.	.
P23M_DN1731_c0_g1_i1	9.90	2.25E-10	Male αα vs. ββ	Adult αα vs. ββ, Female αα vs. ββ	Seaweed composition 2	*Cf-Inv(1)*	.	.	.	.	.	1^20^0.847	.	.	.	.	.	.
P21F_DN1215_c1_g1_i1	11.13	8.26E-15	Male αα vs. ββ	Adult αα vs. ββ, Female αα vs. ββ	Seaweed composition 1, Abiotic characteristics	*Cf-Inv(1)*	.	.	.	.	.	.	.	.	.	.	.	.

Supplemental Table 5 - Abiotic characters/Differential expression overlap. For log2 foldchange Positive values indicate higher expression in ββ and negative values indicate higher expression in αα. if a term was significant in multiple DE analyses we

Transcript	log2 FoldChange	Adjusted *P*-value	Analysis	Additional Analyses where significant	Environmental Association	Physical Location	sprot_Top_BLASTX_hit	sprot_Top_BLASTP_hit	uniprot_sprot.fasta_BLASTX	uniprot_sprot.fasta_BLASTP
P10M_DN1168_c0_g1_i1	-8.77	2.33E-13	Male αα vs. ββ	Adult αα vs. ββ, Female αα vs. ββ	Seaweed composition 1&2, Abiotic characteristics	*Cf-Inv(1)*	.	.	.	.
P10M_DN1478_c0_g1_i2	-7.24	4.44E-09	Male αα vs. ββ	Adult αα vs. ββ, Female αα vs. ββ	Abioitic characteristics	*Cf-Inv(1)*	.	LGR5_BOVIN^LGR5_BOVIN^C	Q80X72^Q80X72^Q:370-1449,H	F1MT22^F1MT22^
pool_DN31577_c0_g1_i1	-7.02	6.93E-06	Female αα vs. ββ	Adult αα vs. ββ	Seaweed composition 2, Abiotic characteristics	*Cf-Inv(1)*	.	.	.	.
P10M_DN38_c0_g1_i5	-6.07	7.80E-29	Female αα vs. ββ	Adult αα vs. ββ, Male αα vs. ββ	Abioitic characteristics	*Cf-Inv(1)*	.	NFH_RAT^NFH_RAT^Q:151-3	P19246^P19246^Q:530-991,H:	P16884^P16884^
P10M_DN38_c0_g1_i10	-8.15	4.63E-08	Male αα vs. ββ		Abioitic characteristics	*Cf-Inv(1)*	.	.	.	.
pool_DN29225_c0_g1_i1	-5.89	2.99E-02	Female αα vs. ββ		Seaweed composition 1, Abiotic characteristics	*Cf-Inv(1)*	.	.	.	.
P10M_DN1345_c0_g1_i4	-5.73	1.54E-03	Male αα vs. ββ	Adult αα vs. ββ, Female αα vs. ββ	Abioitic characteristics	*Cf-Inv(1)*	.	.	.	.
P1M_DN24384_c0_g1_i1	-5.53	9.34E-07	Male αα vs. ββ	Adult αα vs. ββ, Female αα vs. ββ	Abioitic characteristics	*Cf-Inv(1)*	.	.	.	.
P3F_DN5644_c0_g1_i1	-4.02	8.80E-05	Male αα vs. ββ	Adult αα vs. ββ, Female αα vs. ββ	Abioitic characteristics	*Cf-Inv(1)*	.	.	.	.
P13F_DN1815_c0_g1_i2	-3.85	2.11E-07	Male αα vs. ββ	Adult αα vs. ββ, Female αα vs. ββ	Abioitic characteristics	*Cf-Inv(1)*	.	TSN31_MOUSE^TSN31_MOU	Q9CQ88^Q9CQ88^Q:212-856,H	Q9CQ88^Q9CQ88
P10M_DN4982_c0_g1_i1	-3.40	2.55E-02	Male αα vs. ββ		Abioitic characteristics	*Cf-Inv(1)*	.	ECLH_DROME^ECLH_DROME	Q07892^Q07892^Q:282-503,H:	Q07892^Q07892^
P13F_DN3466_c0_g1_i1	-3.28	1.86E-04	Male αα vs. ββ	Adult αα vs. ββ, Female αα vs. ββ	Abioitic characteristics	*Cf-Inv(1)*	OB99A_DROME^OB99A_D	OB99A_DROME^OB99A_DRO	Q9VAJ4^Q9VAJ4^Q:546-878,H:	Q9VAJ4^Q9VAJ4^
P1M_DN2711_c0_g1_i2	-3.11	1.22E-06	Male αα vs. ββ	Adult αα vs. ββ, Female αα vs. ββ	Seaweed composition 2, Abiotic characteristics	*Cf-Inv(1)*	P5CR1_PONAB^P5CR1_PC	P5CR2_BOVIN^P5CR2_BOVIN	Q5R9X6^Q5R9X6^Q:945-1322,I	Q17QJ7^Q17QJ7^
P3F_DN12206_c0_g1_i1	-2.83	5.85E-04	Female αα vs. ββ	Adult αα vs. ββ, Male αα vs. ββ	Seaweed composition 2, Abiotic characteristics	*Cf-Inv(1)*	.	.	.	.
P13F_DN18101_c0_g1_i1	-2.82	4.85E-02	Larvae αα vs. ββ		Abioitic characteristics	*Cf-Inv(1)*	.	CCHL_CHICK^CCHL_CHICK^Q:	Q5F339^Q5F339^Q:191-973,H:	Q5F339^Q5F339^
P3F_DN11202_c0_g1_i1	-2.65	4.98E-02	Male αα vs. ββ		Abioitic characteristics	*Cf-Inv(1)*	SC5A8_HUMAN^SC5A8_H	SC5A8_HUMAN^SC5A8_HUM	Q8N695^Q8N695^Q:227-1891,	.
SKBB_DN2192_c0_g1_i1	-2.51	3.05E-02	Male αα vs. ββ		Seaweed composition 2, Abiotic characteristics	*Cf-Inv(1)*	.	.	.	.
P10M_DN2169_c0_g1_i2	-2.47	2.74E-04	Male αα vs. ββ		Seaweed composition 1, Abiotic characteristics	*Cf-Inv(1)*	.	.	.	.
P3F_DN3410_c0_g1_i4	-2.45	1.47E-02	Male αα vs. ββ		Abioitic characteristics	*Cf-Inv(1)*	YF64_CAEEL^YF64_CAEEL	LTAA_AERJA^LTAA_AERJA^Q:	Q21890^Q21890^Q:111-845,H:	.
P13F_DN2053_c0_g1_i3	-2.15	7.95E-03	Adult αα vs. ββ		Abioitic characteristics	*Cf-Inv(1)*	.	OB99B_DROME^OB99B_DRO	.	Q9VAI6^Q9VAI6^
P13F_DN1192_c0_g1_i9	-2.03	8.01E-03	Male αα vs. ββ		Abioitic characteristics	*Cf-Inv(1)*	DHB13_HUMAN^DHB13_	DHB13_HUMAN^DHB13_HUI	Q7Z5P4^Q7Z5P4^Q:728-1507,H	Q7Z5P4^Q7Z5P4^
P13F_DN1702_c0_g1_i1	-2.00	9.93E-04	Male αα vs. ββ		Abioitic characteristics	*Cf-Inv(1)*	.	TPIS_DROYA^TPIS_DROYA^Q:	O77458^O77458^Q:482-1222,H	O77458^O77458^
P21F_DN1748_c0_g1_i3	2.17	3.32E-03	Male αα vs. ββ		Seaweed composition 1, Abiotic characteristics	*Cf-Inv(1)*	FACD2_HUMAN^FACD2_H	FACD2_HUMAN^FACD2_HUN	Q9BXW9^Q9BXW9^Q:412-440	Q9BXW9^Q9BXW
P23M_DN1144_c0_g2_i2	2.26	3.12E-02	Male αα vs. ββ		Abioitic characteristics	*Cf-Inv(1)*	SC5A7_DROME^SC5A7_D	SC5A7_DROME^SC5A7_DRON	Q9VE46^Q9VE46^Q:1018-2850	.
P13F_DN17181_c0_g1_i1	2.36	3.62E-02	Male αα vs. ββ		Abioitic characteristics	*Cf-Inv(1)*	.	CTR2_ANOGA^CTR2_ANOGA	Q17025^Q17025^Q:136-810,H:	Q17025^Q17025^
pool_DN770_c0_g2_i3	2.73	3.76E-02	Adult αα vs. ββ		Abioitic characteristics	*Cf-Inv(1)*	.	.	.	.
P23M_DN1218_c0_g1_i1	3.16	1.62E-03	Male αα vs. ββ		Abioitic characteristics	*Cf-Inv(1)*	.	.	.	.
P23M_DN2320_c0_g1_i1	3.71	1.77E-07	Male αα vs. ββ	Adult αα vs. ββ, Female αα vs. ββ	Abioitic characteristics	*Cf-Inv(1)*	KCD16_MOUSE^KCD16_M	KCD16_MOUSE^KCD16_MOU	Q5DTY9^Q5DTY9^Q:359-1036,I	.
P21F_DN10984_c1_g1_i1	3.80	1.04E-09	Male αα vs. ββ	Adult αα vs. ββ, Female αα vs. ββ	Abioitic characteristics	*Cf-Inv(1)*	ECSIT_DROME^ECSIT_DRC	ECSIT_DROME^ECSIT_DROM	Q9U6M0^Q9U6M0^Q:330-138	Q9U6M0^Q9U6M
P23M_DN1100_c0_g4_i1	3.92	5.16E-06	Male αα vs. ββ	Adult αα vs. ββ, Female αα vs. ββ	Seaweed composition 1, Abiotic characteristics	*Cf-Inv(1)*	ZG49_XENLA^ZG49_XENL	ZN484_HUMAN^ZN484_HUM	P18724^P18724^Q:394-594,H:	.
P23M_DN639_c0_g1_i7	4.18	1.40E-13	Male αα vs. ββ	Adult αα vs. ββ, Female αα vs. ββ	Abioitic characteristics	*Cf-Inv(1)*	TSN31_PIG^TSN31_PIG^Q	TSN13_HUMAN^TSN13_HUM	Q29257^Q29257^Q:349-576,H:	.
P3M_DN1678_c0_g1_i3	4.96	4.79E-02	Adult αα vs. ββ		Abioitic characteristics	*Cf-Inv(1)*	ABCA3_MOUSE^ABCA3_N	ABCA3_MOUSE^ABCA3_MOU	Q8R420^Q8R420^Q:123-1091,I	.
P23M_DN5697_c0_g1_i1	5.31	1.21E-05	Male αα vs. ββ	Adult αα vs. ββ, Female αα vs. ββ	Abioitic characteristics	*Cf-Inv(1)*	.	.	.	.
P3M_DN3188_c0_g1_i3	5.83	3.32E-02	Male αα vs. ββ		Seaweed composition 1, Abiotic characteristics	*Cf-Inv(1)*	DOCK9_HUMAN^DOCK9_	.	Q9BZ29^Q9BZ29^Q:494-604,H:	.
P21F_DN2360_c0_g1_i1	5.93	2.57E-02	Female αα vs. ββ		Abioitic characteristics	*Cf-Inv(1)*	NPC2_MOUSE^NPC2_MO	NPC2_BOVIN^NPC2_BOVIN^	Q9Z0J0^Q9Z0J0^Q:149-496,H:3	P79345^P79345^
P23M_DN24621_c0_g1_i1	6.20	1.71E-03	Female αα vs. ββ	Adult αα vs. ββ, Male αα vs. ββ	Seaweed composition 2, Abiotic characteristics	*Cf-Inv(1)*	SYMM_DROME^SYMM_D	SYMM_DROME^SYMM_DRO	Q9VFL5^Q9VFL5^Q:1-249,H:46	Q9VFL5^Q9VFL5^
P23M_DN3897_c0_g1_i2	6.23	4.79E-03	Male αα vs. ββ		Abioitic characteristics	*Cf-Inv(1)*	.	.	.	.
P13F_DN2178_c0_g1_i8	6.34	8.82E-04	Larvae αβ vs. ββ		Abioitic characteristics	*Cf-Inv(1)*	.	PPBT_MOUSE^PPBT_MOUSE	P09242^P09242^Q:73-402,H:3	P09242^P09242^
P3M_DN8667_c0_g2_i3	6.44	2.15E-06	Male αα vs. ββ	Female αα vs. ββ, Male αα vs. ββ, Larvae αα vs. ββ	Abioitic characteristics	*Cf-Inv(1)*	LRC15_MOUSE^LRC15_M	LGR5_BOVIN^LGR5_BOVIN^C	Q80X72^Q80X72^Q:256-1032,H	.
P3M_DN9012_c0_g1_i2	9.27	9.77E-11	Male αα vs. ββ	Adult αα vs. ββ, Female αα vs. ββ	Abioitic characteristics	*Cf-Inv(1)*	.	.	.	.
P23F_DN10015_c0_g1_i1	9.29	2.85E-08	Male αα vs. ββ	Adult αα vs. ββ, Female αα vs. ββ	Abioitic characteristics	*Cf-Inv(1)*	.	.	.	.
P23M_DN434_c0_g1_i31	10.82	2.14E-12	Male αα vs. ββ	Adult αα vs. ββ, Female αα vs. ββ	Abioitic characteristics	*Cf-Inv(1)*	SPRR1_RABIT^SPRR1_RAB	.	P35324^P35324^Q:817-1068,H	.
P21F_DN1215_c1_g1_i1	11.13	8.26E-15	Male αα vs. ββ	Adult αα vs. ββ, Female αα vs. ββ	Seaweed composition 1, Abiotic characteristics	*Cf-Inv(1)*	.	.	.	.
P3M_DN722_c0_g1_i11	5.80	7.09E-03	Larvae αα vs. ββ		Seaweed composition 1, Abiotic characteristics	*Cf-Inv(1)*	.	.	.	.

show the data from the most significant test and list that one in the analysis column. The additional tests are listed under ‘Additional analyses where significant’.

Pfam	SignalP	TmHMM	eggnog	Kegg	gene_ontology_BLASTX	gene_ontology_BLASTP	gene_ontology_Pfam
.	.	.	.	.	.	.	.
PF13306.7^LRR_5	sigP:1^21^0.	.	COG4886^le	.	.	GO:0005887^cellul	GO:0005515^molecular_function^protein binding
.	.	.	.	.	.	.	.
PF02389.16^Corni	sigP:1^25^0.	.	ENOG410XPT	.	.	GO:0030424^cellul	.
.	.	.	.	.	.	.	.
.	.	.	.	.	.	.	.
.	.	.	.	.	.	.	.
.	.	.	.	.	.	.	.
.	.	.	.	.	.	.	.
PF00335.21^Tetra	.	.	ENOG4111IR	KEGG:mmu:6	.	GO:0016021^cellul	GO:0016021^cellular_component^integral component of membrane
PF04736.13^Eclosi	.	ExpAA=19.73	ENOG41127V	KEGG:dme:D	.	GO:0005615^cellul	GO:0008255^molecular_function^ecdysis-triggering hormone activity`GO:0007218^biological_process^neuropeptide signaling pathway`GO:0018990^biologica
PF01395.23^PBP_	.	.	ENOG41116	KEGG:dme:D	GO:0012505	GO:0012505^cellul	GO:0005549^molecular_function^odorant binding
PF14748.7^P5CR_	.	.	COG0345^py	KEGG:bta:50	GO:0005739	GO:0005739^cellul	.
PF05649.14^Pepti	sigP:1^28^0.	.	.	.	.	.	GO:0006508^biological_process^proteolysis
PF01265.18^Cyto_	.	.	ENOG410YAU	KEGG:gga:42	.	GO:0005743^cellul	GO:0004408^molecular_function^holocytochrome-c synthase activity`GO:0005739^cellular_component^mitochondrion
PF00474.18^SSF^S	.	.	COG0591^sy	KEGG:hsa:16	GO:0016324	GO:0016324^cellul	GO:0022857^molecular_function^transmembrane transporter activity`GO:0055085^biological_process^transmembrane transport`GO:0016020^cellular_comp
PF00080.21^Sod_	.	.	.	.	.	.	GO:0046872^molecular_function^metal ion binding`GO:0006801^biological_process^superoxide metabolic process
.	.	ExpAA=42.28	.	.	.	.	.
PF01212.22^Beta_	.	.	COG2008^Al	KEGG:ag:BA	GO:0005829	GO:0016829^mole	GO:0016829^molecular_function^lyase activity`GO:0006520^biological_process^cellular amino acid metabolic process
PF01395.23^PBP_	sigP:1^26^0.	.	ENOG4110N	KEGG:dme:D	.	GO:0005576^cellul	GO:0005549^molecular_function^odorant binding
PF00106.26^adh_	.	.	COG1028^De	KEGG:hsa:34	GO:0005829	GO:0005829^cellul	.
PF00121.19^TIM^	.	.	COG0149^tri	.	.	GO:0004807^mole	GO:0004807^molecular_function^triose-phosphate isomerase activity
PF14631.7^FancD	.	.	ENOG410XT	KEGG:hsa:21	GO:0000793	GO:0000793^cellul	GO:0006281^biological_process^DNA repair
PF00474.18^SSF^S	sigP:1^24^0.	.	COG0591^sy	KEGG:dme:D	GO:0016021	GO:0016021^cellul	GO:0022857^molecular_function^transmembrane transporter activity`GO:0055085^biological_process^transmembrane transport`GO:0016020^cellular_comp
PF00089.27^Tryps	.	.	COG5640^pr	KEGG:aga:Ag	.	GO:0005576^cellul	GO:0004252^molecular_function^serine-type endopeptidase activity`GO:0006508^biological_process^proteolysis
.	sigP:1^17^0.	.	.	.	.	.	.
.	sigP:1^42^0.	.	.	.	.	.	.
PF02214.23^BTB_	.	.	ENOG410Z1	KEGG:mmu:3	GO:0042995	GO:0042995^cellul	GO:0051260^biological_process^protein homooligomerization
PF06239.12^ECSIT	.	.	ENOG41100	KEGG:dme:D	GO:0005737	GO:0005737^cellul	.
PF13912.7^zf-C2H	.	.	COG5048^Zi	KEGG:hsa:83	GO:0005634	GO:0005634^cellul	GO:0003676^molecular_function^nucleic acid binding
.	sigP:1^15^0.	.	ENOG4111IR	KEGG:hsa:27	GO:0016021	GO:0005887^cellul	.
PF00005.28^ABC_	.	.	COG1131^(A	KEGG:mmu:2	GO:0097208	GO:0097208^cellul	GO:0005524^molecular_function^ATP binding`GO:0016887^molecular_function^ATPase activity
.	sigP:1^26^0.	.	.	.	.	.	.
.	.	.	ENOG410XN	KEGG:hsa:23	GO:0005829	.	.
PF02221.16^E1_D	sigP:1^17^0.	.	ENOG4111Q	KEGG:bta:28	GO:0005783	GO:0005783^cellul	.
.	.	.	COG0143^Is	KEGG:dme:D	GO:0005759	GO:0005759^cellul	.
.	.	.	.	.	.	.	.
PF00245.21^Alk_p	.	.	COG1785^all	KEGG:mmu:1	.	GO:0031225^cellul	GO:0016791^molecular_function^phosphatase activity
PF00560.34^LRR_	sigP:1^21^0.	.	COG4886^le	KEGG:mmu:7	GO:0009986	GO:0005887^cellul	GO:0005515^molecular_function^protein binding
.	.	.	.	.	.	.	.
.	.	.	.	.	.	.	.
.	sigP:1^17^0.	.	ENOG410XU	.	GO:0005737	.	.
.	.	.	.	.	.	.	.
.	.	.	.	.	.	.	.

. . . . . .

al_process^ecdysis, chitin-based cuticle

ponent^membrane

ponent^membrane

Supplemental Table 6 - Crossing Scheme

Cross type	Population 1	Population 2	# of replicate crosses
αα X ββ	Skeie	Skeie	8
ββ X ββ	Skeie	Skeie	7
αα X ββ	Skeie	Skadbergsanden	5
ββ X ββ	Skeie	Skadbergsanden	5
αα X ββ	Skeie	Smygehuk	5
ββ X ββ	Skeie	Smygehuk	9
αα X ββ	Skeie	Ystad	7
ββ X ββ	Skeie	Ystad	7
